# NAD^+^ Degrading Enzymes, Evidence for Roles During Infection

**DOI:** 10.3389/fmolb.2021.697359

**Published:** 2021-08-16

**Authors:** Arnold Tan, Craig L. Doig

**Affiliations:** Interdisciplinary Science and Technology Centre, Department of Biosciences, School of Science and Technology, Nottingham Trent University, Nottingham, United Kingdom

**Keywords:** NAD^+^, cADPr, PARPs, SIRTs, SARM-1, ARTCs, Infectious disease

## Abstract

Declines in cellular nicotinamide adenine dinucleotide (NAD) contribute to metabolic dysfunction, increase susceptibility to disease, and occur as a result of pathogenic infection. The enzymatic cleavage of NAD^+^ transfers ADP-ribose (ADPr) to substrate proteins generating mono-ADP-ribose (MAR), poly-ADP-ribose (PAR) or O-acetyl-ADP-ribose (OAADPr). These important post-translational modifications have roles in both immune response activation and the advancement of infection. In particular, emergent data show viral infection stimulates activation of poly (ADP-ribose) polymerase (PARP) mediated NAD^+^ depletion and stimulates hydrolysis of existing ADP-ribosylation modifications. These studies are important for us to better understand the value of NAD^+^ maintenance upon the biology of infection. This review focuses specifically upon the NAD^+^ utilising enzymes, discusses existing knowledge surrounding their roles in infection, their NAD^+^ depletion capability and their influence within pathogenic infection.

## Introduction

Fundamental to all cells, the pyridine nucleotide nicotinamide adenine dinucleotide (NAD) was initially identified as “cozymase” at the turn of the 20th Century. Identification of this heat-stable factor present within low molecular weight fractions of yeast that elevated fermentation rates established a platform for subsequent incremental advances (Harden and Young, 1906). As a result, we now understand NAD to be critical in driving hundreds of biochemical reactions as a co-enzyme for energy metabolism. We also better appreciate its contribution towards both individual cell and whole-body homeostasis. NAD itself serves numerous REDOX reactions either via the donation or acceptance of electrons, catalysed by oxidoreductases to dictate the existence of 2 forms: oxidized as NAD^+^, or reduced into NAD(H). The ratio between these 2 species is critical for key energetic processes – for example, in nutrient uptake and energy metabolism, where both forms are utilized for the coupling of the tricarboxylic acid (TCA) cycle and oxidative phosphorylation for adenosine triphosphate (ATP) generation. Additional to REDOX contributions, NAD^+^ acts as a rate-limiting co-substrate for a number of protein and RNA modifying enzymes – primarily, the Poly (ADP-ribose) polymerases (PARPs), Sirtuins (SIRTs) and cyclic ADP Ribose (cADPr) synthases (Xiao et al., 2018; Katsyuba et al., 2020). Recent reports of aberrant NAD^+^ concentrations have been documented in a variety of pathological conditions and in particular, metabolic and age-related dysfunctions (reviewed in Goody and Henry (2018) and Katsyuba et al. (2020)). These data have led to a surge in studies focused on understanding NAD^+^ biology and the ability of precursors to restore NAD^+^ or resist decline of this crucial co-enzyme. In parallel, altered NAD^+^ concentrations have also been ascribed to an increased susceptibility to pathogenic infection and altered immune system response. Here, we review existing and emerging findings surrounding NAD^+^ homeostasis in relation to its utilisation by the post-translational modifying enzymes it rate-limits. Both this role and its contribution to cell biochemistry make adequate intracellular NAD^+^ maintenance an essential influence in sustaining organism health.

## Maintaining NAD^+^ Balance Within the Cell

Given the key nature of NAD^+^ to cell and tissue homeostasis, acquiring an understanding of its formation and breakdown has proven fundamental in improving our understanding of basic metabolic processes and development of disease. Herein, we describe the established pathways of NAD^+^ biosynthesis, salvage and introduce the major enzymatic degraders of NAD^+^ as well as the potentials of NAD^+^ precursor supplementation to treat decline-associated disorders.

### The Biosynthesis of NAD^+^

Three pathways of NAD^+^ biosynthesis within mammals have been identified: Salvage, *de novo*, and Preiss-handler. During basal conditions, the salvage pathway is thought to be the dominant source of NAD^+^. Recycling of the NAD^+^ precursor nicotinamide (NAM) released as a by-product following NAD^+^ degradation, NAM is subsequently converted into nicotinamide mononucleotide (NMN) in a reaction catalysed by NAM phosphoribosyltransferase (NAMPT) (Revollo et al., 2004). NMN is then converted into NAD^+^ by the NMN adenylyl transferases (NMNATs). Nicotinamide riboside (NR), a vitamin B3 precursor of NAD^+^, is also converted into NMN via phosphorylation as part of the nicotinamide riboside kinase (NMRK) 1 and 2 pathways (Bieganowski and Brenner, 2004; Fletcher et al., 2017; Elhassan et al., 2019).

NAD^+^ levels are also found to vary in each subcellular compartment. For example, the 3 isoforms of mammalian NMNATs are localized to different areas of the cell, with NMNAT-1 in the nucleus, NMNAT-2 in the cytoplasm and Golgi apparatus, and NMNAT-3 in the mitochondria (Raffaelli et al., 2002; Zhou et al., 2002; Revollo et al., 2004; Berger et al., 2005 ; Ryu et al., 2018; Okabe et al., 2019). Evidence of NAD^+^ compartmentalization is further supported by the presence of comparable NAD^+^ levels between cytosol and nuclear fractions (Cambronne et al., 2016). Mitochondrial NAD^+^ levels are higher in comparison to those within nuclear and cytosol fractions (VanLinden et al., 2015), and cytosol NAD^+^ production exerts influence over mitochondrial NAD^+^ (Cambronne et al., 2016). Higher mitochondrial NAD^+^ levels have been ascribed to the ability of the mitochondria to uptake NAD^+^ precursors (Cantó et al., 2012; Davila et al., 2018; Klimova et al., 2019) as well as NAD^+^ itself (Davila et al., 2018). The recent identification of a novel mammalian NAD^+^ mitochondrial transporter, SLC25A51, has been confirmed to govern NAD^+^ import into the mitochondria (Kory et al., 2020; Luongo et al., 2020).

The *de novo* and Preiss-handler pathways are dependent on dietary derived vitamin B3 precursors. For the former, referred to as the kynurenine metabolic pathway, tryptophan is converted into quinolinic acid and into nicotinic acid (NA) mononucleotide (NAMN) by quinolinate phosphoribosyltransferases (QPRTs). NMNATs then catalyse the conversion of NAMN into nicotinate adenine dinucleotide (NaAD) which is finally converted by NAD synthases (NADSs) into NAD^+^ (Berger et al., 2005). The Preiss-handler pathway utilizes NA, which is converted it into NAMN by nicotinate phosphoribosyltransferase (NAPRTs) (Evans et al., 2010). This NAMN is subsequently converted into NAD^+^ in the same manner as just described. In recent times, evidence has been presented that all 3 NAD^+^ biosynthesis pathways can switch between (Zhang et al., 2019) and compensate for one another (Okabe et al., 2019).

Importantly, there is a need to establish the difference between transient NAD^+^ availability and absolute repletion through NAD^+^ precursor supplementation. Modest changes to NAD^+^ abundance produced by NR can translate to dramatic improvements to cellular bioenergetics (Liu L. et al., 2018). Within any sample, these modest changes measured over the average tissue can underlie more profound shifts inside an individual cell. Therefore, NAD^+^ turnover as an important cellular function stands distinct from absolute NAD^+^ abundance. This also underpins the value of measuring NAD^+^ utilisation on a tissue-specific basis and highlights that measurement of absolute NAD^+^ levels cannot fully reflect its dynamic flux or biological influence upon a given tissue.

### Major NAD^+^ Degrading Enzymes and Their Roles

Whilst NAD^+^ is appreciated to be fundamental for a cell, it is the flux of NAD^+^ that represents more of a challenge to further our understanding. For example, ratios between NAD^+^:NAD(H), rates of turnover in energy utilisation and insults to genome integrity can induce either transient or sustained fluctuations. This plasticity compounds efforts to understand the consequences of shifts in NAD^+^:NAD(H) ratios. Moreover, NAD^+^ serves as a crucial determinant of the abundance of other species of NAD such as the reduced form of NAD phosphate (NADP(H)). Alongside NAD(H), NADP(H) pools play crucial roles in REDOX maintenance, particularly as antioxidant cofactors (reviewed in Ying, 2008 and Xiao et al., 2018). Since NAD^+^ is the precursor for NADP(H), its decline may impinge NADP(H) levels, altering NADP^+^:NADP(H) ratios due to mitochondrial electron chain dysfunction and inflammation (Bradshaw, 2019). However, reduced forms of NAD^+^ can demonstrate contrasting resilience, and NADP(H) levels remain constant regardless of fluctuating intracellular NAD^+^ levels (Liu L. et al., 2018). Additionally, preservation of NAD^+^ in the form of NADP(H) (for example by NAD kinase) serves to sustain REDOX homeostasis. Importantly, the levels of the reduced form, NADP^+^ can themselves influence the activity of NAD^+^ consuming enzymes (Bian et al., 2019). The distinct compartmentalised pools within cells allow effective sharing of NAD^+^ whilst protecting metabolic utilisation of NAD^+^/NADH within essential TCA cycle and electron transport chain functions.

Nevertheless, significant changes in NAD^+^ as well as its precursors and derivatives, have been reported in studies pertaining to infection. In this section, we describe the major NAD^+^ degrading enzymes and discuss findings of their involvement in overall homeostasis resulting from their utilization of NAD^+^.

#### PARPs

ADP-ribose (ADPr) was first described in the late 1960s as a product possessing the moieties of phosphate and ribose following incubation with the NAD^+^ precursor NMN (Chambon et al., 1963; Chambon et al., 1966; Fujimara et al., 1967; Nishizuka et al., 1967). This work was succeeded by the discovery of the multi-domain containing PARPs and their ability to apply multiple ADPr groups onto target proteins as a post-translational modification, whilst degrading NAD^+^ to yield NAM (Doly and Petek, 1966; Nishizuka et al., 1967; Nishizuka et al., 1968; Ueda et al., 1968). Covalent attachment of monomeric ADPr units occurs on Thr, Glu, Asp, Lys, Arg, Cys or Ser residues of target proteins, shifting biological activity (Vyas et al., 2014). However, PARP-mediated ADP-ribosylation is also applied to DNA and RNA, increasing the opportunities by which NAD^+^ can be degraded (Weixler et al., 2021). Attachment of a single ADPr, known as mono-ADP-ribosylation (MARylation), serves as an initiation step and can be succeeded by the subsequent elongation and branching of poly-ADPr (PAR) chains, known as poly (ADP-ribosyl)ation (PARylation). PARylation chains involve the formation of ribose-ribose linkages between MAR units, usually joined together by a 2″,1‴-glycosidic bond. As each MAR or PAR is derived from a single NAD^+^ molecule, excessive MARylation and consequently, PARylation, represents a substantial drain on intracellular NAD^+^. PARylation is also reversible and regulated through the rapid catalytic degradation of PAR chains by the enzymes PAR-glycohydrolase (PARG) and ADP-ribosylhydrolase 3 (ARH-3) (Davidovic et al., 2001; Niere et al., 2012). PAR chains are also stabilized by macrodomain-containing proteins – for example, MacroH2A1.1 works to prevent rapid PAR chain degradation and avoid excessive NAD^+^ consumption (Ruiz et al., 2019). While excess NAD^+^ degradation by PARPs was initially thought to exacerbate cellular death due to bioenergetic failure (Berger et al., 1983), this cell death mediated by PARP activity was subsequently discovered to exhibit features of necrosis, ascertaining an involvement for PARPs in intrinsic cell death pathways rather than bioenergetic failure alone (Virág et al., 1998). Parthanatos, a cell death pathway dependent upon PAR chain formation, works independent of caspase activity and has been attributed to PARP-mediated NAD^+^ depletion (reviewed in David et al., 2009 and Robinson et al., 2019). Recently, the histone PARylation factor 1 (HPF-1) has been identified as an integral modulator of PARP-1 PARylating activity as part of the DNA damage response (Gibbs-Seymour et al., 2016; Suskiewicz et al., 2020; Rudolph et al., 2021). Such work has provided novel insights into the factors that modulate rates of NAD^+^ degradation by PARP-1 and has implications for the future development of PARP inhibition as a clinical strategy. Together, these actions are largely rate-limited by NAD^+^ availability underscoring both the value of this essential cofactor and its utilisation by PARPs as crucial determinants of cell fate during challenges of genetic stress and pathogenic infection.

Humans and mice have 17 and 16 members of the PARP family respectively. The majority of PARP activity, basal or stimulated, is exerted by either PARP-1 (approximately 85–90%) or PARP-2 (approximately 10–15%). Due to their roles in DNA repair and transcriptional control, their presence is concentrated within the nucleus. Cytoplasmic and mitochondrial PARP activity, however, have also been described (Bai, 2015; Héberlé et al., 2015; Liu L. et al., 2018; Xu et al., 2019), as is the corroborated presence of NAD^+^-dependent ADPr in the mitochondria (Hopp et al., 2021). The recent confirmation that mitochondrial NAD^+^ levels can influence PARP-1 nuclear activity also indicate NAD^+^-mediated mitochondrial-nuclear crosstalk (Hopp et al., 2021). As a result, PARPs are seen as one of the dominant enzymatic consumers of NAD^+^ within the cell. Thus far, out of the 17 PARPs, PARP-1, PARP-2, PARP-5a (Tankyrase 1) and PARP-5b/6 (Tankyrase 2) have been demonstrated to exhibit PARylating activity whilst the remaining members exhibit MARylation activity with the exception of PARP-13 which is catalytically inactive (Langelier et al., 2018). PARylating members possess characteristic and highly conserved residues – H862, Y896 and E988, which form the His-Tyr-Glu (HYE) triad signature critical for NAD^+^ binding and subsequently, PARylating activity (Hassa and Hottiger, 2008; Alemasova and Lavrik, 2019). PARPs also auto-PARylate to regulate intrinsic activity. The influence of PARP-mediated PARylation is far-reaching in terms of NAD^+^ balance – this, as we will outline in the later sections of this review, extends beyond genome maintenance and into immune response activation and the mechanisms of pathogenic infectious disease (Vastag et al., 2011; Heer et al., 2020).

#### SIRTs

SIRTs derive their name from the Silent Information Regulator 2 (SIR-2), first identified in the late 1970s in *Saccharomyces cerevisiae* (Klar and Fogel, 1979), with subsequent homologs later identified (Ivy et al., 1986). The discovery of SIR-2 being regulated by NAD^+^ availability (Tanny et al., 1999) and able to mediate deacetylation of proteins (Landry et al., 2000) re-galvanised interest in SIRTs and NAD^+^. Moreover, SIR-2 overexpression prolonged lifespan in yeast (Kaeberlein et al., 1999), laying the foundations for NAD^+^ biochemistry being perceived as relevant to health across organism lifespan. Since then, 7 mammalian SIRTs have been identified which are ubiquitously expressed and often localized to specific compartments. All SIRTs possess a conserved catalytic domain entailing a NAD^+^ binding pocket (Yuan & Marmorstein, 2012). Generally, SIRTs catalyse target protein deacetylation at Lys residues through the hydrolysis of NAD^+^ to yield O-acetyl-ADPr (OAADPr) and NAM alongside the resulting deacetylated substrate. OAADPr can be subsequently hydrolysed by ARH-3 into ADPr (Kasamatsu et al., 2011). Like ADPr, OAADPr accumulation can also modulate homeostatic processes including chromatin and transcription regulation as well as Ca^2+^ signalling through interaction with binding partners such as the transient receptor potential melastatin-related channel 2 (TRPM-2) and MacroH2A1.1 (Tong & Denu, 2010). Aside from protein deacetylation activity, SIRT family members also possess alternate catalytic activities such as MARylation that is exerted by SIRT-1, SIRT-4 and SIRT-6, as well as long chain deacylation, exhibited by SIRT-4, SIRT-5, SIRT-6 and SIRT-7 (reviewed in Teixeira et al., 2020). Although SIRTs have a lower affinity for NAD^+^ in comparison to PARPs, SIRTs still play both diverse and essential roles in the integration of immune response, energy metabolism and inflammation.

#### cADPr Synthases

Realisation that pyridine nucleotides could stimulate Ca^2+^ release distinct from endoplasmic reticulum localised IP-3 activation first alluded the presence of further unidentified NAD^+^ driven enzyme(s) (Clapper et al., 1987). Later studies identified and characterised the responsible family as the NAD^+^ degrading cADPr synthases (Lee et al., 1994; Jacobson et al., 1995; Lee et al., 1995). Since this discovery, cADPr synthases have been heavily associated with the mobilisation and regulation of intracellular Ca^2+^. cADPr synthases catalyse formation of cADPr from NAD^+^, cleaving it to yield salvageable NAM and ADPr. In mammals, CD38 is the dominant cADPr synthase and is ubiquitously expressed as a transmembrane bi-functional protein that is both a receptor and an enzyme. Expression of CD38 increases in proportion to chronological age. This, and its position on the surface of immune cells, such as Natural Killer (NK) and T cells (Funaro et al., 1993; Muñoz et al., 2008), has piqued interest in potential immunological roles. As a multi-functional enzyme, CD38 behaves as a NAD^+^ glycohydrolase (NADase), generating ADPr from NAD^+^ or as a hydrolase by converting cADPr into ADPr. In the presence of NA, CD38 can also mediate the conversion of NADP into NA-ADP via a base-exchange reaction. Despite being the major contributor of cADPr activity in mammalian cells, only a minor amount of total product produced by CD38 following NAD^+^ incubation is cADPr, whereas ADPr comprises the majority (Guedes et al., 2020). However, its utilization of 100 NAD^+^ molecules to generate a single cADPr molecule renders CD38 a significant NAD^+^ consumer in mammals (Kim H. et al., 1993; Zielinska et al., 2004). This means activation of cADPr synthases as a result of infection has the potential to induce a rapid decline in cellular NAD^+^ concentrations, rendering the cell susceptible to ATP decrease and energy depletion. Importantly, CD38 also gatekeeps NR and NMN degradation before entry into the cell (Escande et al., 2013; Camacho-Pereira et al., 2016; Tarragó et al., 2018).

The CD38 homolog CD157, is a member of cADPr synthases that also exhibits NADase activity. Unlike CD38, CD157 is significantly weaker in terms of cADPr synthesis efficiency (Hussain et al., 1998) and is predominantly expressed within tissues of the gut and lymphoid (Hernández-Campo et al., 2006). Like CD38, CD157 is implicated in an array of immunomodulatory processes, notably the migration, adhesion and extravasation of leukocytes for recruitment to infection sites during immune activation and is also a marker for myeloid cell differentiation (Todd et al., 1985; Seki et al., 2001).

Additional regulators of cellular NAD^+^ availability with cADPr activity include the sterile alpha and Toll/Interleukin-1 receptor (TIR) motif-containing 1 (SARM-1) protein. A recently identified NAD^+^ degrading enzyme with relevance to infection, SARM-1 is mainly expressed in neurons. However, its TIR domain is able to partake in Toll-like receptor (TLR) signalling for modulation of immune response (Carty et al., 2006; Peng et al., 2010). The TIR domain also entails NAD^+^ utilization to generate ADPr and NAM, as well as cADPr. This process of NAD^+^ cleavage by SARM-1 manifests into initiation of axon destruction during neuronal injury (Essuman et al., 2017; Chen et al., 2021), and destruction is attenuated by *SARM-1* deletion (Geisler et al., 2019; Viar et al., 2020; Chen et al., 2021). Supplementation of NR or nicotinic acid riboside (NAR), as does the overexpression of NAMPT or NMNAT, combats SARM-1-mediated declines in NAD^+^ (Gerdts et al., 2015; Chen et al., 2016; Liu H.-w. et al., 2018).

#### Ecto-ADPr Transferases

Ecto-ADPr transferases (ARTCs) degrade NAD^+^ in a similar fashion as PARPs for the transfer of ADPr groups specifically, onto the Arg residues of target proteins (Glowacki et al., 2002). ARTC-1 was the first mammalian ARTC identified within the rabbit skeletal muscle as a membrane-associated ARTC (Zolkiewska et al., 1992). Subsequently, 5 mammalian ARTCs have been identified. Humans however, possess a pseudo ARTC-2 gene, thereby expressing only 4 of the ARTCs as a result, while murine ARTC-2 exists in 2 allele variants ARTC-2.1 and ARTC-2.2 (Glowacki et al., 2002). ARTCs 1–4 are ecto-enzymes anchored via a glycosylphosphatidylinositol tail to the outer membrane, and ARTC-5 is secreted to the extracellular space (Glowacki et al., 2002). ARTCs 1, 2 and 5 possess a characteristic Arg-Ser-Glu (RSE) triad signature which have been corroborated to exert NAD^+^ binding and Arg-specific MARylation activity while ARTC-3 and ARTC-4 do not possess this triad, rendering them catalytically inactive (reviewed in Hottiger et al., 2010 and Di Girolamo and Fabrizio, 2019).

Amongst the catalytically active ARTCs, ARTC-1 and ARTC-2 are the most well-studied. ARTC-1 is predominantly expressed in airway cells as well as in skeletal and cardiac muscle (Paone et al., 2002; Paone et al., 2006; Leutert et al., 2018). Leutert *et al* recently demonstrated within skeletal and cardiac muscles that ARTC-1 basally MARylates the Arg residues of a number of proteins on the cell surface and extracellular space associated with cell adhesion, muscle contraction, and regulation of signal transduction, and its functional significance was underscored with *ARTC1* ^*−/−*^ mice exerting muscle weakness (Leutert et al., 2018). ARTC-1 also has roles in endoplasmic reticulum stress response via the MARylation of glucose-regulated protein of 78 kDa/immunoglobulin heavy-chain-binding protein (GRP78/BiP) (Fabrizio et al., 2015). Other substrates of ARTC-1, including integrin α7, fibroblast growth factor 2 (FGF-2) and platelet-derived growth factor-BB (PDGF-BB) have been identified (Zolkiewska and Moss, 1993; Boulle et al., 1995; Jones and Baird, 1997; Saxty et al., 2001).

In contrast, the murine ARTC-2, initially identified as RT-6 as an alloantigen, is expressed within T cells, NK cells and macrophages (Wonigeit et al., 1997; Okamoto et al., 1998; Hong et al., 2009). This, together with studies of ARTC-1 in airway cells controlling inflammatory response, strongly imply roles for these catalytically active ARTCs in immunomodulatory response which will be further discussed in *ARTCs* of this review. To date, the functional characterisation of ARTC-5 and its associated substrates remains relatively elusive. Nevertheless, the presented studies suggest that ARTCs exert dynamic influence over different aspects of physiology.

## Host Cell NAD^+^ Degradation During Immune Activation

**FIGURE 1:**
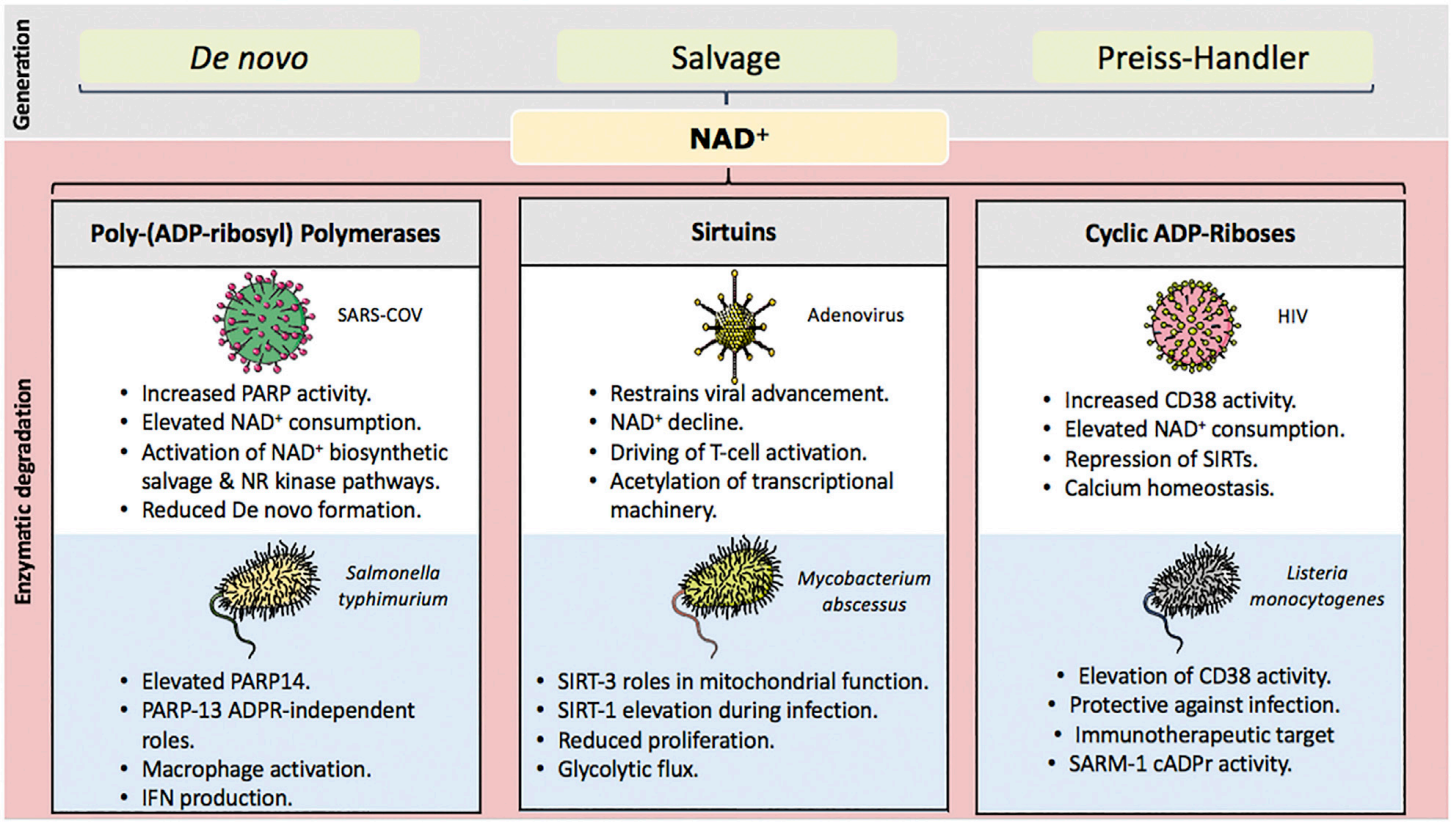
Key NAD^+^ degrading enzyme and their influence over infection. NAD^+^ is generated through three pathways *de novo*, biosynthetic salvage and their Preiss-handler pathway. NAD^+^ consuming enzymes yield nicotinamide for salvage and conversion back into NAD^+^. Their activation during infection represents a significant challenge for the host cell. Chronic declines in NAD^+^ concentations are a key mediator of both immune and metabolic dysfunction. Approaches to sustain and/or replete celluar NAD^+^ through precursor supplementation have considerable potential to lessen detrimental outcomes during times of infection.

The intrinsic value of vitamin B3-derived NAD^+^ to human health is best demonstrated by its depletion in the nutritional condition pellagra. Absence of the essential amino acid tryptophan and vitamin B3 from dietary sources produces profound disease including characteristic dermatitis (photosensitive), diarrhoea, dementia and death. However, accumulating evidence also suggests that NAD^+^ consumption is central to both mechanisms driving pathogenic infection and the host cell’s capability to resist infectious disease (Figure 1). Here, we outline key implications of the above-described NAD^+^ degrading enzymes in relation to the immune system in terms of cellular response to infection, initiation and advancement of inflammatory response.

### PARPs

Historically, PARP activity as a regulator of inflammatory response was proposed three decades ago where expression of pro-inflammatory cytokines such as Tumour Necrosis Factor alpha (TNF-α) and Interleukin 6 (IL-6) were downregulated in macrophages following PARP inhibition (Pellat-Deceunynck et al., 1994). *PARP-1* ^*−/−*^ mice exhibit inflammatory resistance to Zymosan-induced inflammation (Szabó et al., 1997). Initiation of inflammation promotes PARP-1 nuclear translocation by Nuclear factor kappa B (NF-κB). This drives expression of other cytokines including Interferon-γ (IFN-γ) and TNF-α, contributing to the inflammatory response (Oliver et al., 1999; Shou et al., 2019). PARP-1 also co-regulates transcriptional activation of these pro-inflammatory factors: Nuclear factor of activated T-cells (NFAT) (Valdor et al., 2008), Activator protein 1 (AP-1) (Andreone et al., 2003), and Yin Yang 1 (YY-1) (Oei and Shi, 2001). Regulation by PARP-1 is also exerted through its ubiquitin-mediated degradation of IκB kinase, subsequently elevating NF-κB activity (Hinz et al., 2010).

In addition to activation of key mediators of immune response, PARP-1 also differentially regulates immune cell maturation and function via Interleukin 10 (IL-10) and Interleukin 12 (IL-12) (Aldinucci et al., 2007). The VDJ recombination phase (or antigen receptor gene rearrangement) that occurs during B and T cell development is also influenced by PARP activity. Here, PARP-1 contributes towards immunoglobulin class switching. Demonstrated in the B-cell lymphoma mouse cell line I.29µ and splenic B cells, PARP inhibition increased IgA switching (Shockett & Stavnezer 1993), while B cells of *PARP-1* ^*−/−*^ mice exhibit aberrant recombination and subsequent immunoglobulin class switching following LPS stimulation, as well as impaired T-cell dependent responses (Ambrose et al., 2009). Sufficient recombination and class switching generates diverse antibodies for antigen recognition, crucial in the defence against pathogens. However, the rate-limiting contribution of NAD^+^ and PARP-1 activity in these processes warrants further investigation. The NAD^+^ status of many of these models and its flux during these experiments still needs to be ascertained. Replication of these studies complete with accurate measurement of NAD^+^ and its associated metabolites would reveal the extent to which PARP-1 immune roles are being governed by NAD^+^ concentrations and if these are a limiting factor at any point.

In contrast, it seems the immune status of the host can also determine the role played by PARP-1. For example, in T cells of mice with severe combined immunodeficiency, deletion of PARP-1 rescued aberrant VDJ recombination associated with the disease (Morrison et al., 1997). Thymocytes, cells prior to thymopoiesis which develop into mature T cells, express both PARP-1 and PARP-2. However, only *PARP-2* ^*−/−*^ mice consequently exhibit reduced overall population numbers of double positive CD4^+^/CD8^+^ thymocytes, suggesting that in this example PARP-1 is dispensable during thymocyte development (Yélamos et al., 2006; Nicolas et al., 2010; Navarro et al., 2017). Consequently, dual deletion of *PARP-1* and *PARP-2* in mice is detrimental to T cell maturation and their mediated immune response (Navarro et al., 2017), establishing the basis for highly coordinated PARP-1 and PARP-2 functionality in T cell as well as B cell development (Galindo-Campos et al., 2019). Importantly, as NAD^+^ and its associated metabolites were not evaluated in these studies, it is unclear if ablations of PARP-1/2 in these models produce T or B cell-specific NAD^+^ increase. Additionally, any resultant surplus of NAD^+^ is liable to utilisation by cADPr synthases or SIRTs. Still, this demonstrates that adequate supply of NAD^+^ for PARP-1 and PARP-2 function is a major determinant in T and B cell development making an adequate supply of NAD^+^ for their proper function critical.

In parallel, PARP-14 drives B cell function through activation of Interleukin 4 (IL-4). This increases expression of B cell survival factors including proto-oncogene serine/threonine-protein kinase (Pim-1) and myeloid-cell leukaemia 1 (Mcl-1), resisting caspase-induced apoptosis (Cho et al., 2009). PARP-14 also serves a bi-functional switch, being a transcriptional co-activator of STAT-6 driving IL-4-mediated transcriptional activation, but repressive in the absence of STAT-6 (Mehrotra et al., 2011). These actions contribute towards excessive T-helper (Th) 2 (Th2) cell-mediated immune response and can promote cell survival through c-Jun N-terminal Kinase 2 (JNK-2) and PGI/AMF signalling (Schweiker et al., 2018), a key event in chronic inflammatory disorders associated with imbalanced immune system function. Macrophages rely on *de novo* NAD^+^ biosynthesis for basal activity and more so during immunity challenge (Minhas et al., 2019), and NAD^+^ degradation by PARP-14 may be contributory in dictating macrophage activity through MARylation of STAT-1. In contrast, utilisation of NAD^+^ by PARP-9 counterbalances pro-inflammatory actions of PARP-14. MARylation of STAT-1 by PARP-14 inhibits STAT-1 phosphorylation, decreasing macrophage activation and tempering inflammatory responses (Iwata et al., 2016). This suggests substantial interplay between ADP-ribosylation and phosphorylation for macrophage activation, further underscoring NAD^+^ as a molecule with far-reaching implications for immune response. This additionally presents coordination in balancing NAD^+^ maintenance in relation to activation of multiple PARPs which can act as dual regulators of both macrophage activation and inhibition (Iwata et al., 2016). Both macrophages and monocytes also upregulate NAMPT during LPS-mediated inflammation (Schilling et al., 2012; Cameron et al., 2019), elevating NAD^+^ formation to meet demand by the cell during immune activation. In accordance with this notion, PARP activity is also significantly upregulated in LPS-stimulated macrophages (based on increased total PAR levels), supporting increased NAD^+^ consumption (Cameron et al., 2019; Minhas et al., 2019). These works highlight the roles and interplay between different NAD^+^ degrading PARP enzymes within the same superfamily in order to achieve immune system balance and proportionality of response. However, how NAD^+^ availability for PARP activity consequently affects overall function, particularly in the context of T and B cell development, requires further investigation.

### SIRTs

Evidence over the last decade has assigned NAD^+^-driven SIRT activity as being immunoregulatory with its roles spanning the entire field of immunology ranging from cell development to chronic inflammatory disorders (Kong et al., 2012). SIRT-1 dictates T cell immune response – specifically, CD8^+^ T cells, Th and regulatory T (Treg) cell differentiation. T cell tolerance via interaction with c-Jun and CD4^+^ T cell inhibition and terminally differentiated memory T cell metabolic reprogramming occurs through a SIRT-1/FOXO-1 axis mechanism (Jeng et al., 2018). In addition to T cell regulation, SIRT-1 also modulates self-renewal in macrophages via regulation of cell cycle progression and self-renewal gene network (Imperatore et al., 2017). Recently, the overexpression of SIRT-1, confirmed to reduce NAD^+^ availability, promoted B cell viability (Gan et al., 2020). Encouragingly, the activation of SIRT-1 was also shown to be tempered by reducing NAD^+^ concentrations, emphasising the critical nature of NAD^+^ abundance to ensure sufficient immune activation.

In addition to SIRT-1, SIRT-2 has been shown to be activated by immune stimulus, with it shown to be involved in CD8^+^ T cell regulation in tumour immune response (Jiang et al., 2020), as well as Th2 and Th17 cell-mediated immune response (Elesela et al., 2020). Other SIRTs exhibit a differing immune profile – for example, SIRT-3 is dispensable for immune cell development (Ciarlo et al., 2017). While multiple roles for SIRT-1 dynamically impacting host cell NAD^+^ levels have been demonstrated, evidence of SIRT-mediated NAD^+^ decline are limited. While the majority of evidence surrounding SIRT-1 activity is shown to be inflammatory protective (Pfluger et al., 2008), studies in the context of some immune system disorders have shown otherwise. For example, SIRT-1 promotes the survival of B cell chronic lymphocytic leukaemia cells (Bhalla & Gordon, 2016).

SIRT-1 was also first discovered to deacetylate NF-κB at the p65 subunit to attenuate NF-κB-dependent gene expression (Yeung et al., 2004). However, antagonistic crosstalk exists between both SIRT-1 and NF-κB to switch between innate immunity and energy metabolism pathways (Kauppinen et al., 2013). For one, NF-κB activity stimulates glycolytic pathways for NAD^+^ production that consequently results in SIRT-1 activity which then subsequently represses NF-κB activity and attenuates inflammation, and pharmacological SIRT-1 activation augments this phenomenon (Yang et al., 2012). Mechanistically, these involve players associated with oxidative energy production which act in association with SIRT-1, including AMP-activated protein kinase (AMPK), peroxisome proliferator-activated receptor alpha (PPARα) and peroxisome proliferator-activated receptor gamma coactivator 1-alpha (PGC-1α) (Yang et al., 2010; Planavila et al., 2011).

Studies have also ascertained roles for SIRT-6 in the inflammatory response – Van gool *et al* demonstrated SIRT-6 activity exerted pro-inflammatory properties via the upregulation of TNF-α expression in murine bone marrow-derived dendritic cells, which was abolished upon the depletion of intracellular NAD^+^ or SIRT inhibition (Van Gool et al., 2009). Similar observations for NAD^+^-dependent SIRT-6 involvement in production of other pro-inflammatory cytokines including IFN-γ and Interleukin 8 (IL-8) in immune cells have also been described (Bruzzone et al., 2009; Montecucco et al., 2013). In contrast, using tissues of *SIRT-6* ^*−/−*^ mice, it was demonstrated that SIRT-6, like SIRT-1, was able to associate with the NF-κB p65 subunit to subsequently entail the deacetylation of H3 histone lysine 9 (H3K9) and consequently attenuating NF-κB signalling. This suggests SIRT-6 may have an anti-inflammatory role which could be a contributing factor in the lethality of *SIRT-6* ^*−/−*^ mice (Kawahara et al., 2009). Additionally, a recent study in human umbilical vein endothelial cells ascribed SIRT-6 as anti-inflammatory via modulating expression of nuclear factor erythroid 2 related factor 2 (NRF-2), a transcription factor regulating expression of antioxidant proteins. Mechanistically, the knockdown of SIRT-6 reduced TNF-α-induced NRF-2 expression, whereas knockdown of NRF-2 significantly enhanced TNF-α-induced expression of the pro-inflammatory cytokines MCP-1, IL-6 and interleukin 1 beta (IL-1β). Subsequently, SIRT-6 overexpression dampened expression of these cytokines, suggesting protective roles for SIRT-6 against vascular inflammation via its deacetylase activity and the NRF-2-dependent signaling pathway (He et al., 2021). Henceforth, these studies of SIRT-6 suggest that its role as pro or anti-inflammatory could be dependent on the type of inflammation at play.

Together, these works highlight the fundamental nature of NAD^+^ availability to inflammatory response and the value of strategies such as NAD^+^ precursor supplementation that are able to protect against NAD^+^ depletion. Importantly, SIRT-1 regulates immunometabolism via the regulation of Hypoxia-inducible factor 1 alpha (HIF-1α), entailing proper cytokine production and immune cell differentiation, bridging together innate and adaptive immune response (Yu D. et al., 2018).

### cADPr Synthases

As stated earlier, CD38 governs T cell responsiveness and leukocyte adhesion through cADPr-mediated Ca^2+^ signalling (reviewed in Audrito et al., 2019). However, elevated expression may be also be responsible for the deprivation of NAD^+^ and subsequent loss of the protective activity of SIRT-1-mediated T cell homeostasis. *CD38* ^*−/−*^ mice secrete reduced monocyte chemoattractant protein 1 (MCP-1) following cerebral artery occlusion, ablating inflammation due to altered immune cell migration and subsequently activation (Choe et al., 2011). Moreover, CD38 expression is thought to be mediated by both TNF-α and NF-κB (Kang et al., 2006) and extracellular NAD^+^ potentiates granulocyte recruitment to inflammatory sites due to increased cADPr synthase activity and subsequently enhanced Ca^2+^ signalling (Bruzzone et al., 2006). Immune activation by LPS elevates CD38 surface expression in both monocytes and macrophages (Schilling et al., 2012; Cameron et al., 2019), implicating it in a key role for NAD^+^ dependent inflammatory response to cytokine release and immune cell senescence (Covarrubias et al., 2021). Like CD38, CD157 has exerted similar roles in immune system function through regulation of B and T cell development and inflammatory response via mediating myeloid cell migration and extravasation (reviewed in Ortolan et al., 2019 and Glaria and Valledor, 2020).

In contrast, while the TIR domain of SARM-1 mediates TLR signalling for immune response function, it is a negative regulator of both TRIF and MyD88-dependent TLR signalling (Luo et al., 2019) and holds NAD^+^ hydrolase activity. However, SARM-1 mediates the clearance of activated and expanded clones of T cells following immune challenge – whereby its expression is elevated to dampen pro-survival cascades via inhibition of ERK phosphorylation and downregulation of the expression of the anti-apoptotic protein Bcl-xL. Subsequently, this disrupts mitochondrial integrity resulting in activation of the pro-apoptotic factors caspase-9 and caspase-3 to initiate apoptosis for T cell clearance (Panneerselvam et al., 2013). While studies surrounding SARM-1 are primarily focused within the nervous system, inflammatory response in the brain mediated by SARM-1 has been described. SARM-1 regulates the expression of pro-inflammatory cytokines including TNF-α, IL-6, MCP-1, RANTES, IFN-α, IFN-β and MIP-1 (Hou et al., 2013). This suggests SARM-1 could link infection and neuronal injury by incorporating NAD^+^ availability in this role (Gerdts et al., 2015; Essuman et al., 2017; Chen et al., 2021).

### ARTCs

Immunomodulatory effects of ARTC-mediated MARylation were first described in murine T cell lymphoma EL4 cells, where ARTC-1 overexpression MARylates distinct cell surface molecules associated with T cell receptor signalling including CD43, CD44, CD45 and lymphocyte function-associated antigen 1 (LFA-1), resulting in impaired signalling arising from failure of T cell receptors and co-receptors to associate into a contiguous and functional receptor cluster (Liu et al., 1999). ARTC-1 also MARylates human neutrophil peptide 1 (HNP-1), a family member of α-defensins – these are Arg-rich peptides secreted by immune cells and are implicated in anti-microbial innate immunity response (Paone et al., 2002). Indeed, Paone *et al* have published 2 excellent studies unravelling the functional significance of ARTC-1-mediated MARylation of HNP-1 – first, MARylation of HNP-1 at Arg-14 modifies its biological properties resulting in decreased anti-microbial and cytotoxic activities while concomitantly elevating IL-8 release in contrast to unmodified HNP-1. Furthermore, HNP-1, regardless of modification status, exerts influence as a chemoattractant for the recruitment of T cells (Paone et al., 2002). The second study corroborated roles for ARTC-1 in inflammatory response – levels of ADP-ribosylated HNP-1 were observed to be elevated within the bronchoalveolar lavage fluid of patients with respiratory inflammatory diseases such as pulmonary fibrosis and asthma, as well as in regular smokers (Paone et al., 2006), implying that MARylation of HNP-1 by ARTC-1 is part of the inflammatory response in the airway. These works underscore the therapeutic potential of ARTC-1 activity in treatment of respiratory diseases.

In contrast, expression of the murine ARTC-2 on the surface of immune cells suggests roles for ARTC-2 in immunity – notably, the process of NAD^+^-induced cell death (NICD) in T cells via the MARylation of the Arg-125 residue on the purinergic receptor P2X7, a member of the P2X receptor family of ATP-gated ion channels. Seman *et al* demonstrated that millimolar concentrations of ATP, as does micromolar concentrations of NAD^+^ (1 µM), are sufficient for ADP-ribosylation and subsequent activation of the P2X7 receptor by ARTC-2 (Seman et al., 2003). However, given that extracellular concentrations of NAD^+^ in serum is between 0.1–0.3 µM (Kim U. H. et al., 1993), it suggests a mechanism whereby ARTC-2 remains inactive basally and traumatic events such as inflammation and tissue injury release NAD^+^ into the extracellular space to activate ARTC-2-mediated MARylation and activation of the P2X7 receptor. Subsequently, activation initiates a cascade of events including increased calcium flux, exposure of phosphatidylserine and breakdown of mitochondrial membrane potential to ultimately result in T cell death by apoptosis (Seman et al., 2003). Additionally, the extent of NICD is proportionate to the levels of P2X7 receptor expression – for example, Treg cells which express higher levels of P2X7 than effector T cells are more susceptible to NICD (Nemoto et al., 1996; Kahl et al., 2000), and T cells of *ARTC-2* ^*−/−*^ mice are resistant to NICD (Seman et al., 2003). Furthermore, 2 receptors for immunoglobulin, FcγR1 and FcγR2B have been identified as substrates of ARTC-2-mediated MARylation within the murine microglia, implying roles for ARTC-2 in central nervous system immunity (Rissiek et al., 2017). Additionally, an involvement of ARTC-1 in P2X7 receptor signalling has been studied, suggesting altered function and expression of P2X7 and ARTC-1 in human CD39^+^ Treg or CD39^−^ Treg cells could participate in the resistance against cell death induced by ATP or NAD^+^ (Cortés-Garcia et al., 2016).

## Evidence for NAD^+^ Degrading Enzymes in Pathogenic Infection

During infection, host cells typically generate dynamic metabolic signatures invoked as a consequence of pathogens competing for host nutrients and hijacking of homeostatic pathways. Metabolic reprogramming of host cells due to pathogen-mediated manipulation of pathways have been well-described (reviewed in Eisenreich et al., 2019). Such shifts commonly comprise of elevated glucose uptake and glycolytic flux, reflecting increased demand (Diray-Arce et al., 2020). Variances in carbohydrate metabolism are also likely to mediate shifts in NAD(H) levels – for example, important studies have identified the targeting of NAD^+^ generating pathways by pathogens to result in metabolic disruption (Grady et al., 2012; Heer et al., 2020).

Overall loss of NAD^+^ cell homeostasis significantly impinges pathogen clearance. However, dynamic changes in NAD^+^ concentrations once infection has become established seemingly differ. Although these too, can be pathogen-specific, as well as host cell-specific. As an example, viruses such as herpes simplex virus (HSV), human immunodeficiency virus (HIV), zika virus (ZIKV) and severe acute respiratory syndrome coronavirus 2 (SARS-CoV2) severely deplete the host NAD^+^ pool, mostly due to over-activation of PARPs. This works to favour replication while concomitantly depleting or circumventing immune cell response (Murray et al., 1995; Grady et al., 2012; Minhas et al., 2019; Xu et al., 2019; Heer et al., 2020). In such cases, there is a clear rationale for the elevation of NAD^+^ levels by supplementation as a strategy to mitigate detrimental impacts of increased enzymatic degradation (Heer et al., 2020; Migaud et al., 2020).

Bacterial pathogens, such as *Plasmodium* and *Leishmania* are shown to elevate host cell NAD^+^ pools through divergent mechanisms. For example, human erythrocytes infected with *Plasmodium* exhibit increased NAD^+^ synthesis from NAM and NA by targeting the elevation of NAMPT and NAPT activity (Zerez et al., 1990), whilst *Leishmania infantum*-infected macrophages exert a transient increase in NAD(H) before reverting to increased NAD^+^/NAD(H) ratios following infection establishment (Moreira et al., 2015). However, divergent effects on NAD^+^ status within the same classes of pathogens have also been observed. For example, human cytomegalovirus (HCMV) infected cells do not cause a decline in NAD^+^. This, in comparison to HSV infection which depletes host cell NAD^+^, demonstrates contrasting strategies in altering host cell metabolism to favour their replication and survival (Vastag et al., 2011). Consequently, the contrasting metabolic strategies exhibited by both pathogens resulted in differing responses to treatments, where inhibition of fatty acid synthesis impairs HCMV replication but not HSV (Vastag et al., 2011). Similarly, Group A Streptococcus such as *Streptococcus pyogenes* exhibits strong NADase activity, severely depleting host intracellular NAD^+^ levels (Michos et al., 2006). This results in dysfunction of autophagy and phagolysosome acidification, in addition to increasing PARP-1 activity to activate inflammation and ultimately cell death (Hsieh et al., 2018). Similar mechanisms whereby NAD^+^ depletion is exerted to impair the pathogenic clearance response are also observed in *Mycobacterium tuberculosis* (Pajuelo et al., 2018). Further investigation to understand the pathways by which NAD^+^ influences metabolic strategies amongst pathogens work to favour persistence and infection are needed.

### Roles of NAD^+^ Degrading Enzymes During Pathogen Infection

#### PARPs

##### Basis for PARP Involvement in Pathogenic Infections

Roles for PARPs in pathogenic infection are well-established but the importance of NAD^+^ availability to this process is only now being fully appreciated. Recent findings surrounding responses to viral infection show PARP-1 regulates recruitment of NK cells to infected sites through NF-κB-mediated MCP-1 production (Shou et al., 2019). This further supports NAD^+^ availability for consumption by PARPs as a central unit in anti-viral defence. PARPs 4, 9, 13, 14 and 15, have been reported to undergo recurrent evolutionary processes, including positive selection for renewal of novel adaptations to pathogenic infection, making ADP-ribosylation a crucial determinant in the outcome of host-virus interaction (Daugherty et al., 2014; Gossmann & Ziegler, 2014). Stress granules, composed of aggregates of proteins and RNA within the cytosol, can form during infection – these contain heavily PARylated proteins, Tankyrase 1 and PARPs 12 to 15, as well as PARG (Leung et al., 2011). It is likely that accumulation of stress granules represents a significant pressure upon NAD^+^ homeostasis by PARPs and probable scenarios for infection-related NAD^+^ depletion. Severe depletion of NAD^+^ during most pathogenic infections mainly arises due to targeted over-activation of PARPs, (primarily PARP-1). Because of their role as mediators of pro-inflammatory pathways, persistent PARP-1 activation and consumption of intracellular NAD^+^ subsequently causes bioenergetic failure which manifests into PARP-1-mediated inflammation in the form of cell necrosis, exacerbating cell death (Hsieh et al., 2018; Xu et al., 2019).

Some viral families, mainly Coronaviruses, Togaviruses and Herpesviridae, possess macrodomain proteins that bind both MAR and PAR units, hydrolysing them. Studies have since corroborated the functional significance in tandem with the structural characterization of these macrodomains in modulating PARP catalytic activity to mediate viral replication and pathogenesis (Li et al., 2016; Abraham et al., 2018; Grunewald et al., 2019a,b; Alhammad and Fehr, 2020; Rack et al., 2020; Schuller et al., 2021). These studies not only strongly implicate PARPs as a modulatory target that can be utilised by pathogens during infection to evade immune response and drive replication, they also underscore the significance of these viral macrodomains as targets for pioneering therapeutic strategies to combat pathogenicity, particularly in the context of SARS-CoV2 which has been recently presented (Rack et al., 2020; Schuller et al., 2021). However, the interaction with NAD^+^ elevating nutritional strategies remains to be resolved. For example, there is a clear need for evidence from infected patients demonstrating reduced NAD^+^ levels, elevated PARP activity and amelioration by NAD^+^ supplementation.

##### PARP-1 and Tankyrase Expression and Activity are Modulated During Pathogenic Infection

Several studies have demonstrated NAD^+^-dependent PARP activity as a target during infection. For example, the mosquito-borne ZIKV can severely deplete intracellular NAD^+^ through activation of PARP-1, which consequently interacts with the ZIKV NS3 protein to mediate cell death. This effect was abolished when PARP-1 was knocked down (demonstrated with rescued NAD^+^ concentrations), signifying the value of PARP-1 to ZIKV (Xu et al., 2019). Gastric mucosa infected by *Helicobacter pylori* exhibit elevation in PARP-1 that resulted in overactivation of NF-kB-mediated inflammation (Lee et al., 2016). *Trypanosoma cruzi* infection of cardiomyocytes upregulates activity of the ROS–PARP-1–p65 pathway, entailing persistent cytokine production to culminate in chronic inflammation (Ba et al., 2010). *Salmonella enterica* serovar Typhimurium infection of epithelial cells also enhanced PARP-1 and PARP-2 expression particularly in the nucleus (Qi et al., 2017). Similarly, HSV-1 infection elevates PARP-1 and PARP-2 activity again reducing NAD^+^ levels and mediating ubiquitin-proteasomal degradation of PARG through the ICP-0 viral protein. However, importantly, the knockdown of all PARG isoforms significantly reduced viral titres, suggesting a mechanism for fine-tuning the regulation of auto-PARylation-mediated inhibition of PARP-1 and PARP-2 (Grady et al., 2012). This corroborates earlier findings showing PARP activity as being essential for HSV-1 replication, where expression of Tankyrase 1 was elevated during HSV-1 infection and viral titres were substantially reduced following knockdown of Tankyrases 1 and 2 (Li et al., 2012). Furthermore, PARP-1 and Tankyrase 1 PARylate the Epstein Barr Virus (EBV) protein EBNA-1 to consequently destabilise the viral episome in latently infected cells due to inhibition of EBNA-1 binding at the dyad symmetry elements of OriP, implying anti-viral roles (Deng et al., 2005; Tempera et al., 2010). Corroborating this, HCMV infection of human foreskin fibroblasts induced the expression of Tankyrases 1 and 2 while concomitantly reducing its PARylation activity to consequently drive viral replication, as does shRNA-mediated knockdown of both (Roy et al., 2015). In contrast, Influenza A Virus (IAV) infection of A549 cells was recently demonstrated by Bamunuarachchi *et al* to also elevate Tankyrase 2 expression. However, its shRNA-mediated knockdown reduced IAV viral titres, suggesting pro-viral roles. Mechanistically, the authors identified Tankyrase 2 as a target of the anti-viral microRNA miR-206 which consequently inhibits IAV replication and activates Type I IFN immune response (Bamunuarachchi et al., 2021).

Retroviruses, and in particular HIV-1, have proven to be more contentious when identifying roles for PARP-1. Work applying loss-of-function strategies to PARP-1 via genetic knockout (Ha et al., 2001; Kameoka et al., 2005; Gutierrez et al., 2016), pharmacological inhibition (Rom et al., 2015; Piekna-Przybylska et al., 2019) or siRNA silencing (Kameoka et al., 2004) all document a dependency upon PARP-1 for HIV-1 genomic integration. However, other studies have posited a dispensable role for PARP-1 in the similar process (Siva and Bushman, 2002; Ariumi et al., 2005; Bueno et al., 2013), and even the conferment of resistance to HIV-1 infection (Bueno et al., 2013). Potential reasons for these contrasting findings include functional redundancy with 17 members of the PARP family in mammals, where the loss-of-function in PARP-1 might be compensated by other members, and background model strain as intra-experimental variables. Additionally, there are species-specific events to consider – for example, contrasting results between mammalian and avian cells pertaining to HIV-1 integration have been observed (Bueno et al., 2013). Once successful viral integration has occurred, PARP-1 can work in assistance to the viral unit. For example, it has been reported that PARP-1 can modulate transactivation of HIV-1 Tat protein which subsequently results in HIV-1 long terminal repeat promoter activity and initiation of the HIV-1 transcriptional program (Rom et al., 2015; Yu H.-B. et al., 2018). In further support of its pro-viral roles, Wang *et al* demonstrated HIV-1 superinfection further elevates PARP-1 expression (Wang et al., 2017). These works suggest chronic HIV-1 viral load levels are a consequence of a greater drive of HIV-1 transcriptional program during latent infection and utilises PARP-1. In contrast to working in union with host cell PARP, the E4orf4 protein of Adenoviruses is recruited by PARP-1 and associates with it to DNA damaged sites whereby it inhibits PARP-1 activity. This occludes DNA damage response whilst concomitantly amplifying replication (Nebenzahl-Sharon et al., 2019). Furthermore, purpose for PARP-1 in driving infection is found by the function of the IAV RNA-Dependent RNA polymerase, which is dependent on PARP-1. IAV inhibits PARylating activity and further amplifies viral replication. However, pharmacological inhibition of PARP-1 PARylating activity drives replication, indicating that NAD^+^ concentrations are unlikely to be profoundly impacted, and implicates PARP-1 being a facilitator rather than an essential meditator for IAV replication (Westera et al., 2019). Xia *et al* recently supported this notion using A549 cells and demonstrating IAV viral hemagglutinin interacts with PARP-1 to degrade type I IFN receptor 1 (IFNAR-1), subsequently inhibiting antiviral IFN response, entailing viral propagation (Xia et al., 2020). HSV-1 and ZIKV genome integration and replication occur independently of PARP-1 activity (Grady et al., 2012; Xu et al., 2019), while the replication of the John Cunningham virus (JCV) is suppressed in the presence of the selective PARP-1 inhibitor, 3-aminobenzamide (Nukuzuma et al., 2013). Distinctively, anti-viral functions of PARP-1 have also been described – PARP-1 inhibits the pre-integration step of avian retrovirus infection (Gutierrez et al., 2016), the transcriptional program of hepatitis B virus (HBV) (Ko et al., 2013), lytic replication of the EBV by binding to the lytic switch BZLF-1 promoter (Lupey-Green et al., 2017), as well as Kaposi’s sarcoma-associated herpesvirus (KSHV) (Cheong et al., 2015). Collectively, all of the studies suggest that PARP-1 and the Tankyrases playing roles as pro or anti-viral is partly determined by the type of infectious agent. Crucially, given PARP-1’s role in the DNA damage response and chromatin modification, it could suggest that viruses with a DNA genome stage have their replication modulated by PARP-1. However, investigation into the dynamics of NAD^+^ consumption and how it impacts PARP-1 pro or antiviral activity are avenues worth pursuing as is the potential for NAD^+^ supplementation as a therapy.

##### Dynamic Influence of the Remaining PARPs During Pathogenic Infection

The influence of the other NAD^+^ dependent PARPs is seemingly more dynamic during pathogenic infection, though this view may be more a consequence of the decreased knowledge surrounding their roles and levels of activity. For example, SARS-CoV2 induces expression of several noncanonical PARPs – PARP-7, PARP-9, PARP-10, PARP-11, PARP-12, PARP-13 and PARP-14 were substantially upregulated following infection with the magnitude of subsequent NAD^+^ depletion being proportional with viral titres (Heer et al., 2020). Outcomes from SARS-CoV2 infection have been associated to an individual’s overall health and concomitant underlying pathologies having influence, particularly type II diabetes and morbid obesity. Pro-inflammatory markers regulated by PARPs such as TNF-α and IL-6 have been observed to be upregulated during infection that contribute towards the cytokine storm during infection (Curtin et al., 2020). Infection is inherently stressful and NAD^+^ degrading enzymes play prominent roles for both the host and infectious agent. PARP-7 (TCDD-inducible PARP) is a MARylating PARP and possesses pro-infectious and host-mediated attributes usually within a pathogen-dependent context. Using its zinc finger domain, it suppresses sindbis virus (SINV) replication by binding to and mediating degradation of SINV viral RNA (Atasheva et al., 2014; Kozaki et al., 2017), albeit by an undefined mechanism. PARP-7 also drives IAV replication by NAD^+^-driven ADP-ribosylation of TANK binding kinase 1 (TBK-1), and this suppresses IFN production (Yamada et al., 2016). PARP-7 knockdown reduces murine coronavirus (MHV) viral titres in murine bone-marrow-derived macrophages (BMDMs) (Grunewald et al., 2019b).

PARP-9 is another of the MARylating PARPs whose activation is established following heterodimerization with Dtx3L, a histone E3 ligase involved in the DNA damage response (Zhang et al., 2015; Yang et al., 2017). This PARP-9-Dtx3L heterodimer subsequently exerts anti-viral effects by ubiquitinating the histone protein H2BJ to promote expression of IFN-stimulated genes (ISGs) which subsequently abrogate IAV, SINV and encephalomyocarditis virus (EMCV) replication. Moreover, PARP-9-Dtx3L heterodimer ubiquitinates EMCV viral 3C protease, resulting in its degradation and disruption of viral assembly (Zhang et al., 2015). PARP-9 also drives the phosphorylation of STAT-1 and pro-inflammatory markers, as well as IFN-γ-mediated responses in murine and human macrophages. These processes are all co-ordinated through PARP-14 expression and activity, demonstrating the arrangement between different PARPs working to mediate viral and host responses (Iwata et al., 2016). Transcriptome-wide association study by Han *et al* identified *PARP-9* as a novel marker for the prognosis of HBV infection, with expression of *PARP-9* increased in response to HBV infection (Han et al., 2020). Furthermore, the promoter region of *PARP-9-Dtx3L* was identified within a cluster of differentially methylated regions in genome-wide association studies of HIV-1 infection. This suggests HIV-1 induces genome-wide methylated changes in *PARP-9* to evade immune response (Oriol-Tordera et al., 2020). Further studies are necessary to ascertain mechanisms of PARP-9 in viral replication and the potential impact of PARP-9 activation during infection. It also remains to be established if NAD^+^ concentrations flux at any point during infection with HIV-1.

A MARylating PARP, PARP-10 possesses an IFN-stimulated response element/virus response element (ISRE/VRE) within its promoter. This becomes activated in response to infection by the Newcastle Disease Virus (NDV) (Mahmoud et al., 2011) and IFN stimulation (Grunewald et al., 2019a). The PARP and Glu-rich domains of PARP-10 are targeted by avian H5N1 influenza virus NS1 viral protein to repress PARP-10 expression and consequently drive replication (Yu et al., 2011). PARP-10 also influences the replication of Venezuelan equine encephalitis virus (VEEV) by inhibiting cellular translation (Atasheva et al., 2012). The dominant influence of PARP activity over cellular NAD^+^ levels is eloquently shown in a recent examination using PARP-10 overexpression. This was sufficient to deplete cellular NAD^+^ levels as robustly demonstrated in HEK293 cells (Heer et al., 2020).

Recent data have also implicated PARP-11 as a mediator of infection. Overexpression in Vesicular stomatitis virus (VSV), HSV-1 and IAV infection during infection, PARP-11 MARylates the ubiquitin E3 ligase β-transducin repeat-containing protein (β-TrCP). This results in proteasomal degradation of IFNAR-1, dampens IFN-1 signalling and overall anti-viral response (Guo et al., 2019). Additionally, this work also demonstrates the efficacy of the PARP inhibitor Rucaparib towards PARP-11 – inhibition of which *in vivo* attenuates VSV and HSV-1 infection by reducing viral replication and enhancing IFN-1 signalling. These studies identify PARP-11 as a potential target with utility in the treatment of some viral infections.

PARP-12L (Long isoform) has been demonstrated with PARP-7, to inhibit VEEV replication via impairment of translation (Atasheva et al., 2012). This provides additional anti-viral roles of PARP-12L working in combination with PARP members. PARP-12L was also found to MARylate NS1 and NS3 viral proteins to mark these viral proteins for ubiquitin-mediated proteasome degradation, impairing ZIKV replication (Li L. et al., 2018). Whilst PARP-12 exhibits only MARylating activity, these viral proteins were also observed to be PARylated. This indicates overlapping influence of the PARylating PARPs, and primarily PARP-1 given its physical interaction with NS3 in a later study (Xu et al., 2019). Given the high likelihood of interaction by PARP-1, it is probable that PARP-1 is able to directly PARylate NS3, though this still remains to be ascertained. Similarly, Grunewald *et al* demonstrated siRNA-mediated knockdown of PARP-12 in BMDMs, as well as PARP pharmacological inhibition, were each significant in rescuing impaired viral replication in a mutant MHV virus lacking a functional viral ARH domain (Grunewald et al., 2019a). Given the functional importance of such domains present on a multitude of viruses including MHV (reviewed in Alhammad & Fehr, 2020), the findings delineate a crucial anti-viral role for PARP-12 catalytic MARylating activity which warrants further mechanistic investigation.

Of the noncanonical PARPs, PARP-13, despite lacking catalytic activity, is the most well-described in relation to pathogenic infection. Also known as the Zinc Finger anti-viral protein (ZAP), it has 4 isoforms; ZAP-L, ZAP-S, ZAP-M and ZAP-XL (Li et al., 2019). Recently, it has been reported the alternative splicing of ZAP isoforms is regulated by the ubiquitin E3 ligase, TRIM-25 in an unknown mechanism (Lin et al., 2020). Mechanistically, ZAP-L and ZAP-S partake in the anti-viral defence in contrasting manners. It was found that ZAP-S was capable of binding to host IFN mRNA at the 3’ UTR region, resulting in degradation and dampening of IFN response as a negative feedback regulator of IFN response that occurs after immunity challenge, while ZAP-L exhibited direct anti-viral activity by targeting SINV viral RNA (Schwerk et al., 2019). Nevertheless, ZAP can inhibit the replication of a plethora of viral families including Alphaviruses, Filoviruses, HBV, IAV, Retroviruses, and Arteriviruses (Chemudupati et al., 2019 and references therein; Zhao et al., 2019). Recently, it was demonstrated that ZAP anti-viral activity is further bolstered by interaction with TRIM-25 (Lin et al., 2020). Of note, ZAP targets the RNA of viruses at a specific sequence, known as the ZAP responsive element (ZRE), to subsequently result in RNA processing exosome and poly(A)-specific ribonuclease-mediated degradation (Guo et al., 2007). Recently, it was reported that ZAP expression in stress granules was pertinent for inhibiting the replication of SINV (Law et al., 2019). Indeed, the anti-viral roles of ZAP have resulted in further reports showing its targeting by pathogens to inhibit the function of this protein, including the NS1 viral protein of IAV blocking the binding of ZAP-S to its target ZRE (Tang et al., 2017). Similarly, ZAP protein is cleaved by the 3C protease during enterovirus infection (Xie et al., 2018). Most recently, HCMV has been reported to evade ZAP detection through suppressing CpG dinucleotides, one of the markers of viral infection recognized by ZAP, within its viral immediate early gene 1 (IE1) which is expressed upon infection (Lin et al., 2020).

Similar to PARP-13, PARP-14 has anti-viral influence. In A549 and normal human dermal fibroblast cells, as well as BMDMs, maximal IFN expression in response to coronavirus infection and Polyinosinic:polycytidylic acid stimulation is PARP-14-dependent (Grunewald et al., 2019a). Similarly, *Salmonella typhimurium* infection and proliferation was elevated in PARP-14 CRISPR knockout RAW 264.7 macrophages, suggesting PARP-14 has considerable importance in constraining proliferation (Caprara et al., 2018). This study also demonstrated PARP-14 mediated recruitment of RNA Polymerase II to promoter regions of IFN regulatory transcription factor 3 (IRF-3), enhancing IFN-β production and secondary anti-viral responses. Direct interactions of PARP-14 in restraining pathogen replication remain poorly understood. However, given its critical role in the regulation of IFN production, particularly in Coronavirus infection (Grunewald et al., 2019a), and with IFN therapy being proposed in the context of SARS-CoV2 infection (Sallard et al., 2020), PARP-14 could yet have utility within emerging anti-viral strategies.

These studies show that the dynamics of PARP expression and activity is highly pathogen-specific, with some PARP proteins entirely favouring or suppressing pathogenic infection. In the case of PARP-13, ADP-ribosylating activity, and subsequently NAD^+^ levels, is not the sole determinant of a PARP’s impact over infection. The roles of PARPs 4, 6, 8 15 and 16 to date, have yet to be elucidated in scenarios of pathogenic infection. Nevertheless, these studies presented necessitate further investigation to fully understand how these PARP members are implicated in viral infection – particularly initial stages of integration and successful replication.

#### SIRTs

Given the intrinsic roles SIRTs have as protein deacetylases, a clear principle exists for SIRT involvement during pathogenic infection and the modulation of both pathogen and host gene expression. Emerging evidence suggests a protective role for SIRTs against pathogenic infection which will be presented below.

##### SIRT-Mediated Modulation of Gene Expression in Pathogenic Infection

Using human lung fibroblasts (MRC-5), the siRNA targeted knockdown of 5 out of the 7 members of the mammalian SIRT family resulted in increased viral titres following infection of either HCMV, HSV-1, Adenovirus or IAV. Though notably, knockdown of SIRT-3 and SIRT-7 did not impact viral titre following HSV-1 infection (Koyuncu et al., 2014). In parallel, the pharmacological activation of SIRTs reduced viral titres, further suggesting that SIRT activity maybe important for exhibiting anti-viral effects. These data indicate any anti-viral SIRT activity is rate limited by levels of NAD^+^ – infection by HCMV does not affect NAD^+^ levels, while HSV-1 causes NAD^+^ decline (Vastag et al., 2011). Since its been documented that NAD^+^ levels remain unchanged during HCMV infection, these suggest a mechanism whereby such viruses are capable of evading SIRT activity.

While SIRT-3 and SIRT-7 knockdown did not impact HSV-1 replication, SIRT-3 inhibits HBV replication via deacetylation of H3K9 (Ren et al., 2018), though this this was shown to yield no influence over infectivity (Ciarlo et al., 2017). Moreover, acetylation status of H4 histone lysine 16 (H4K16) that regulates cell cycle progression is deacetylated by both SIRT-1 and SIRT-2 – this was significantly decreased following HCMV infection (O'Connor et al., 2014). This underscores the implication of histone modifications in the anti-viral response of NAD^+^-driven SIRTs.

Mechanistically, targeting SIRT-mediated pathways by viruses results in altered gene expression. The most notable example being HIV, where SIRT-1 deacetylates HIV Tat protein to entail HIV transactivation (Pagans et al., 2005). Intriguingly, the catalytic domain of SIRT-1 is in turn, targeted by Tat to inhibit SIRT-1 activity. This results in enhancement of T cell activation, NF-κB-responsive genes activation and Interleukin 2 (IL-2) production, all of which support viral replication (Kwon et al., 2008). Conversely, treatment using the SIRT activator Resveratrol in tandem with Decitabine (a nucleic acid synthesis inhibitor eliciting anti-HIV activity) has been proposed as a therapeutic option for HIV (Clouser et al., 2012). This combination is conjectured to prevent cycles of Tat acetylation and deacetylation. Castro *et al* demonstrated in both brain capillary pericytes and macrophages that Occludin, a NAD(H) oxidase regulating SIRT-1 activation, is targeted for depletion during HIV infection, increasing NAD(H) levels with concomitant decrease in NAD^+^. This results in nuclear translocation of CtBP1, the SIRT transcriptional repressor, to further dampen activity in tandem with low NAD^+^ and enhance viral replication (Castro et al., 2016). Similarly, the NS1 viral protein of IAV possesses a histone H3-like sequence similar to mammalian H3 histone lysine 4 (H3K4) which is subjected to acetylation capable of impeding anti-viral gene transcription (Marazzi et al., 2012). Together with enhanced IAV replication following SIRT inhibition (Koyuncu et al., 2014), this supports a role for SIRTs in IAV replication. Recently, Xander *et al* reported SIRT-1 to be protective against Rhinovirus (RV)-mediated IFN production and inflammation by repressing JAK/STAT activation in airway epithelial cells. This mechanism is absent in chronic obstructive pulmonary disease (COPD) airway cells due to these cells lacking basal SIRT-1 expression, exacerbating RV-mediated IFN production and chronic inflammation. This can be however, ameliorated by treatment with the SIRT-1 activator Quercetin (Xander et al., 2019).

SIRT-1 is expression is also reduced following *Mycobacterium tuberculosis* infection and global *SIRT-1* ^*−/−*^ mice exhibit an elevated susceptibility to infection. Moreover, Resveratrol administration attenuates infection in acute and chronic *Mycobacterium tuberculosis* infection (Cheng et al., 2017), while SIRT-2 is dispensable in the infection (Cardoso et al., 2015). In contrast, SIRT-1 expression and activity is upregulated in the parasite *Cryptosporidium parvum* (Xie et al., 2014) and in HBV, which subsequently enhances viral replication by driving AP-1-mediated HBV promoter activation. These impacts were abolished following treatment with the SIRT-1 and 2 inhibitor Sirtinol (Ren et al., 2014). Yamai *et al* confirmed that pharmacological SIRT-1 activation using Resveratrol enhanced HBV replication through induction of HBV viral protein HBcAg and pgRNA levels (Yamai et al., 2020). Moreover, Yu *et al* have reported that the selective inhibition of SIRT-2 using AGK2 hindered HBV replication (Yu Q. et al., 2018). Similarly, *SIRT-2* ^*−/−*^ mice exerted resistance against *Listeria monocytogenes* infection due to impaired H3 histone lysine 18 (H3K18) deacetylation that is mediated by SIRT-2 (Eskandarian et al., 2013). KSHV infection can be consequently reactivated with SIRT-1 inhibition, resulting in elevation of activating H3K4me3 and depletion of repressive H3K27me3 histone marks in the RTA promoter (a lytic replication activator) of KSHV (Li et al., 2014). Similarly, SIRT-6 expression is upregulated in RAW 264.7 macrophages following dengue virus infection – mechanistically, the shRNA-mediated knockdown of SIRT-6 exacerbates virus-mediated pro-inflammatory cytokine production including IL-6 and TNF-α via a NF-κB p65 subunit mechanism (Li P. et al., 2018).

Collectively, these suggest SIRTs are a target during pathogenic infection and exploited to sustain viral infection to subsequently determine outcomes of pathogenicity. But again, as with PARPs, there is a clear need to establish NAD^+^ levels and flux (and associated derivatives) during these infections to give a full understanding of how useful modulating SIRT activity *in vivo* may be.

##### Pathogens Target Host Cellular Metabolic Pathways Which Are Regulated by SIRTs

Aside from central functions as regulators of gene expression through their deacetylation roles, NAD^+^-dependent SIRTs exert influence over wider metabolism. Included within these roles are the mitochondrial metabolic processes of oxidative respiration and fatty acid oxidation. The major mitochondrial SIRTs, SIRT-3, 4 and 5 all regulate a variety of critical metabolic processes in the mitochondria. For example, these SIRTs regulate glycolytic and TCA cycles via regulation of key enzymes and substrates, including the pyruvate dehydrogenase complex and malate dehydrogenase (Budayeva et al., 2016). SIRTs are also linked to immunometabolism, such as the metabolic regulation of immune cells, which can consequently affect the outcome of pathogenic clearance (Heinonen et al., 2019; Elesela et al., 2020). Knockdown of these mitochondrial SIRTs results in enhanced viral replication in a variety of viral families (Koyuncu et al., 2014). Mechanistic studies into these effects have been limited, but are emerging: for example, *Mycobacterium abscessus* infection was demonstrated to target SIRT-3 degradation in macrophages to induce mitochondrial dysfunction, ROS production and inflammation. Consequently, SIRT-3 activation using Resveratrol ameliorated these pathophysiological observations (Kim et al., 2020), indicating SIRT-3 preserves energetic pathway function during infection. Similarly, *SIRT-5* ^*−/−*^ mice exerted susceptibility to *Salmonella typhimurium* infection due to impaired glucose homeostasis which subsequently enhanced IL-1β production to exacerbate inflammation (Zhang et al., 2020). While this contradicted results from preceding studies reporting that SIRT-5 has a dispensable role in bacterial infection (Heinonen et al., 2018), mice backgrounds and bacterial strains used in both studies were different, potentially influencing outcomes. However, given the essential nature of NAD^+^ and the cells inherent ability to activate biosynthetic salvage in response to stress, it is likely SIRT-5 has some role to play in both host driven immune processes and infection related processes.

While changes in metabolic status in immune activated cells have been described (reviewed in Preyat and Leo, 2013), emerging evidence demonstrates that pathways such as the TCA cycle and fatty acid oxidation are targeted during pathogenic infection. For example, Vastag *et al* demonstrated through uniformly labelled 13C- glucose that in HCMV infected cells, an additional carbon atom is shuttled through the TCA cycle, resulting in enhanced lipid biosynthesis and this metabolic change was implicated in augmenting HCMV’s ability to replicate (Vastag et al., 2011). Given the role of SIRTs in governing metabolic regulation, as well as the modulation of host metabolic status as a consequence of pathogenic infection, these warrant experimental investigation into SIRTs as a potential target by such pathogens. Yu *et al* presented a model whereby the overexpression of the Hepatitis C virus (HCV) core protein in HepG2 cells degraded SIRT-1 expression and activity, causing REDOX imbalances and decreased intracellular NAD^+^/NAD(H) ratios. As a result, the activity of downstream targets, such as AMPK, are also suppressed, impairing transcriptional regulation and expression of genes associated with fatty acid uptake and oxidation (including PPARα and CPT1). This was exacerbated by concomitant increased expression of fatty acid and triglyceride biosynthesis genes (SREBP-1c, FAS, and ACC) to induce hepatic steatosis as observed during HCV infection (Yu et al., 2013). Similarly, Zhang *et al* reported similar intracellular metabolic perturbations including the infection-mediated hepatic steatosis. However, SIRT-1 expression in contrast was increased and sustained following core protein overexpression. Interestingly, SIRT-1-mediated deacetylation of PPARγ2 and degraded expression of PPARγ2 were also observed. Liver-specific *SIRT-1* ^*−/−*^ mice on the other hand exhibited increased PPARγ2 expression, and lentiviral infection of core protein subsequently attenuated hepatic steatosis (Zhang et al., 2018).

Recently, Sun *et al* also observed downregulation of SIRT-1 following HCV core protein overexpression to consequently activate hepatic stellate cells (Sun et al., 2018). Therefore, while the observations in hepatic metabolic dysfunction following core protein overexpression are irrefutable, the mechanistic disparities in these studies necessitate a remit for further investigation. More specifically, contrasting expression profiles of SIRT-1 following core protein overexpression remains to be reconciled, where Yu *et al* and Sun *et al* describe SIRT-1 degradation following HCV core protein overexpression. As does the degradation of PPARy2, where Yu *et al* reported no significant changes in core protein overexpressed HepG2 cells. Perhaps the SIRT-1-mediated deacetylation of PPARγ2 could be in part due to the interplay between the de/acetylation of target proteins and degradation via the ubiquitin-proteasome system (Hauser et al., 2000). Nevertheless, these studies presented have achieved a consensus for elucidating that HCV-mediated hepatic metabolic dysfunction is ascribed to SIRT-1.

An example whereby SIRTs regulate overall mitochondrial function in immune cells to drive effective immune activation during pathogenic infection was recently described in bone marrow-derived dendritic cells infected with respiratory syncytial virus (RSV). These cells subsequently exhibited mitochondrial dysfunction via disruption of mitochondrial membrane potential, with elevated levels of mitochondrial ROS production (Elesela et al., 2020). Crucially, the mechanisms behind this observation were ascribed to SIRT-1, with the authors of the study demonstrating that *SIRT-1* deletion pre-disposed cells to basal mitochondrial dysfunction and RSV infection consequently exacerbated dysfunction. Resistance to NAD^+^ decline will have presumably been improved with SIRT-1 deletion but this has not yet been demonstrated. SIRT-1 regulates the expression and activity of ACC-1 and FASN, key enzymes involved in fatty acid biosynthesis that were upregulated in *SIRT-1* absence following infection. These were targeted and activated during RSV infection to elevate fatty acid biosynthesis to subsequently cause mitochondrial stress and impaired innate cytokine production. Fundamentally, the presence of SIRT-1 drives the downstream activation of AMPK which then promotes fatty acid oxidation via phosphorylation and inhibition of ACC1 and switching of FASN activity to fatty acid oxidative to subsequently drive appropriate immune response mediated by Th2 and Th17 cells. Given that viral infection and immune defence are reliant on fatty acid metabolism (Thormar et al., 1987; Vastag et al., 2011; Zhang et al., 2018; Heinonen et al., 2019; Ma et al., 2019; Elesela et al., 2020), and how fatty acid biosynthesis/oxidative pathways are regulated by SIRTs and targeted by these pathogens, these studies presented could pave the way forward for SIRT-mediated anti-viral therapy using SIRT activators, particularly in aging individuals with sustained NAD^+^ decline to supplement NAD^+^ boosting therapy. However, diverse metabolic strategies between pathogens must be considered, and the existence of such a phenomenon warrants further study for tailored therapeutics focused on elevating NAD^+^ as a cofactor important to the function of these enzymes.

#### cADPr Synthases

##### CD38 and CD157 Act as Barriers Against Pathogenic Infection

Expression of CD38 on the surface of numerous immune cells indicates its roles in pathogenic infection. This importance is also demonstrated in *CD38* ^*−/−*^ mice which are sensitized to infection by many bacterial pathogens including *Mycobacterium* and *Listeria monocytogenes* due to tempered neutrophil recruitment and inflammatory response (Wei et al., 2014; Glaria and Valledor, 2020). Though as ever, it is important to consider this impact may be pathogen-specific as it was not observed in the context of *Listeria monocytogenes* infection (Lischke et al., 2013). Shifts in CD38 expression have also been observed during infection, although reports of corroborating NAD^+^ concentrations or altered NAD^+^ turnover are scarce. For example, *Listeria monocytogenes* and *Mycobacterium tuberculosis* infection upregulates CD38 (Lischke et al., 2013; Adekambi et al., 2015), as well as in RSV-infected monocyte-derived dendritic cells (MDDCs) in an IFN-dependent manner (Schiavoni et al., 2018) and in HCMV patients (Booiman et al., 2017). CD38 expression in CD8^+^ T lymphocytes have been proposed as a marker for HIV disease progression and treatment outcome (Maurya et al., 2019), although historically, CD38 elevated expression is not directly affected by HIV infection (Savarino et al., 2000). However, the mechanistic influence of CD38 catalytic activity on HIV replication and subsequent pathogenesis remains to be unravelled. What is clear however, is that CD38 is a key consumer and is able to significantly reduce both NMN and NAD^+^ during immune activation (Rodríguez-Alba et al., 2019; Chini et al., 2020).

CD38 serves as a downstream target of the Liver X receptor (LXR), crucial for amelioration of *Salmonella Typhimurium* induced pathologies following treatment with a LXR agonist (Matalonga et al., 2017). In MDDCs, treatments with Kuromanin, a CD38-specific inhibitor, and/or 8-Bromo-cADPr (8-Br-cADPr), a cell-permeant cADPr antagonist blocking cADPr-mediated Ca^2+^ signalling, abolished upregulation of RSV-induced pro-inflammatory genes such as *IFN-β* and *RANTES* without any impact on RSV replication (Schiavoni et al., 2018). This indicates that CD38 catalytic activity and cADPr production are mediators of protection against RSV infection. Similarly, Katsuyama *et al* reported elegant cross-talk mechanisms between NAD^+^ degrading enzymes in patients of the autoimmune disorder, systemic lupus erythematosus. Patients possessed high populations of aberrantly elevated expression of CD38 in CD8^+^ T lymphocytes, and the high CD38 levels depleted intracellular NAD^+^ levels resulting in repressed SIRT-1 activity to elevate acetylated status of its substrate EZH2 and consequently impair transcriptional programmes associated with T lymphocyte cytotoxicity response to amplify susceptibility to pathogenic infections (Katsuyama et al., 2020). This crosstalk mechanism has also been proposed as a model for hyperinflammatory responses observed in SARS-CoV2 and subsequent mortality in aged patients (Miller et al., 2020). Collectively, while these suggest that inhibition of catalytic CD38 activity might be useful in anti-viral therapy, caution should be exercised given CD38 activity is critical for Ca^2+^ signalling that is imperative for other bodily processes and its expression is still pertinent for protection against pathogens (Wei et al., 2014; Nam et al., 2020). This is evident in patients undergoing anti-CD38 antibody mediated immunotherapy, where increases in opportunistic pathogenic infection in such patients have been reported (Glaria and Valledor, 2020).

In contrast, reports on CD157 function during pathogenic infection is relatively limited, despite its expression in immune cells such as neutrophils and macrophages. Only recently, Yang *et al* demonstrated that its expression is imperative for the conferment of host resistance against *Mycobacterium tuberculosis* infection – while CD157 expression is upregulated in circulatory monocytes during infection, repercussions in the lungs of infected *CD157* ^*−/−*^ mice are associated with exacerbated bacterial replication and inflammation. Mechanistically, CD157 expression in macrophages drives TLR2-dependent cytosolic ROS production for the conferment of bactericidal capacity and subsequently, the efficient killing of *Mycobacterium tuberculosis*, which was severely impaired in *CD157* ^*−/−*^ mice. This suggested that these observations were mediated by the receptor function of CD157 rather than cADPr synthase activity. Furthermore, this phenotype was rescued upon exogenous application of soluble CD157, highlighting a potential therapeutic for therapy against this infection (Yang et al., 2019). However, further study regarding the mechanisms of soluble CD157 and TLR2 signalling for effective killing of *Mycobacterium tuberculosis* by macrophages is warranted.

##### SARM-1 is a Meditator of Neuronal Protection in Response to Pathogenic Infection

Although SARM-1 is predominantly expressed on neurons and studies discussed earlier in *cADPr Synthases* ascribe SARM-1 as a driver of neuronal damage, its documented roles in appropriate immune system development and response, alongside the existence of pathogens capable of invading the central nervous system such as *Neisseria meningitidis* and encephalitis-inducing viruses such as HSV make SARM-1 a potential mediator of neuronal injury and death that arise from such infections. However, studies of SARM-1 in this context remains limited.

Melioidosis, the infection of macrophages arising from the bacteria *Burkholderia pseudomallei*, is dependent on SARM-1. Specifically, *in vitro* SARM-1 expression is elevated directly following bacterial internalization, although the process of internalization is independent of SARM-1. Subsequently, siRNA-mediated knockdown of SARM-1 bolsters the bactericidal capacity in macrophages for *Burkholderia pseudomallei* clearance via the upregulation of IFN-β and inducible nitric oxide synthase (iNOS) (Pudla et al., 2011). Similarly, the zoonotic encephalitic La Crosse Virus (LCV) induces *in vivo* SARM-1 upregulation and mitochondrial translocation in cortical neurons due to viral-mediated activation of the mitochondrial anti-viral signalling protein pathway to consequently contribute to apoptotic death – while *SARM-1* ^−/−^ did not directly impact viral replication, neuronal death was strikingly reduced (Mukherjee et al., 2013), suggesting that SARM-1 is a mediator of neuronal death as part of the pathology of LCV infection. In contrast however, SARM-1 deficiency exacerbates the replication of West Nile Virus (WNV) and neuronal death in the brainstem of infected mice without significant impact on replication (Szretter et al., 2009), while Hou *et al* demonstrated that VSV infection is substantially attenuated in *SARM-1* ^−/−^ mice, also without impacting replication (Hou et al., 2013). Mechanistically, *SARM-1* ^*−/−*^ mice infected with VSV and WNV depleted expression of pro-inflammatory cytokines. However, lethality was markedly different between both. Furthermore, mortality in other brain regions aside from the brainstem such as the hippocampus were relatively unaffected in WNV infected *SARM-1* ^*−/−*^ mice (Szretter et al., 2009). These findings suggest that immune responses mediated by SARM-1 are pathogen-specific, as well as tissue-specific, where morbidity might either be associated as a repercussion of direct viral damage or immune-mediated damage as a consequence of infection and clearance response mediated by SARM-1. Collectively, all of the studies presented do achieve a consensus for SARM-1 implication in pathogens capable of invading the central nervous system. However, given its function as a cADPr synthase, further investigation into its catalytic activity in response to fluctuating NAD^+^ dynamics that occur as a result of pathogenic infection could further understand susceptibility to exacerbated pathologies arising from pathogenic infection in the brain in the aging individual.

#### ARTCs

##### ARTCs Functional Impacts in Pathogenic Infection Remain Unclear

Knowledge surrounding both the mechanistic and biological function of ARTCs remains incomplete. However, the extracellular positioning of the extracellular NAD^+^ binding catalytic site is likely to be fundamental to any actions in pathogen-related infection or resistance. Moreover, their capacity to utilise available NAD^+^ remains to be established, as does levels of expression and activity during infection. Once such measures are established, the ARTCs are likely to emerge as novel targets for therapeutic intervention.

## Concluding Remarks

Excessive consumption of cellular NAD^+^ damages attempts to maintain homeostasis. During immune challenge, activation of NAD^+^ degrading enzymes serve as a fundamental driver of NAD^+^ depletion. This is particularly relevant in respect to PARPs and the cADPr synthases as the physiological repercussions of their elevated activity in cells are well-documented. Attempts to counteract this response to physiological stress include the use of NAD^+^ precursor boosting, and human supplementation studies have been carried out with a diverse array of outcomes, often dependent upon the precursor used. In this respect, particular success has been shown with the compounds NR and NMN – whilst some disparity remains between them and their attributed roles within physiology, improvements to health have been achieved.

Outcomes being dependent upon the specific NAD^+^ precursor suggests that there are more biochemical mechanisms of NAD^+^ generation yet to be understood. Most recent discoveries include that of the reduced forms of NR (NRH) and NMN (NMNH) (Giroud-Gerbetant et al., 2019; Zapata-Pérez et al., 2021). Whilst the identification and impact of these NAD^+^ precursors was anticipated, it is important to the wider field and provides exciting new avenues for leverage of NAD^+^ biology to human health. In parallel, the response of NAD^+^ degrading enzymes to supplementation may not be as linear as expected. As an example, the important recent discovery surrounding PARP-1 molecular functions have revealed that it can switch between PARylating and PAR hydrolysis activity via a HPF-1-dependent mechanism (Rudolph et al., 2021). Importantly, both of these processes readily consume NAD^+^, exemplifying potential importance for our conception of PARPs as pure NAD^+^ consumers and indicates a more sophisticated model of PARylation and its biological roles. In light of this, it is apparent that the ability to sustain NAD^+^ flux in response to degrading enzyme(s) activation during immune challenge is critical to both the host immune response and in many cases, the mechanisms driving infection.

## References

[B1] Abraham R. Hauer D. McPherson R. L. Utt A. Kirby I. T. Cohen M. S. (2018). ADP-ribosyl-binding and Hydrolase Activities of the Alphavirus nsP3 Macrodomain Are Critical for Initiation of Virus Replication. Proc. Natl. Acad. Sci. USA 115, E10457–E10466. 10.1073/pnas.1812130115 30322911 PMC6217424

[B2] Adekambi T. Ibegbu C. C. Cagle S. Kalokhe A. S. Wang Y. F. Hu Y. (2015). Biomarkers on Patient T Cells Diagnose Active Tuberculosis and Monitor Treatment Response. J. Clin. Invest. 125, 1827–1838. 10.1172/JCI77990 25822019 PMC4598074

[B3] Aldinucci A. Gerlini G. Fossati S. Cipriani G. Ballerini C. Biagioli T. (2007). A Key Role for Poly(ADP-Ribose) Polymerase-1 Activity during Human Dendritic Cell Maturation. J. Immunol. 179, 305–312. 10.4049/jimmunol.179.1.305 17579050

[B4] Alemasova E. E. Lavrik O. I. (2019). Poly(ADP-ribosyl)ation by PARP1: Reaction Mechanism and Regulatory Proteins. Nucleic Acids Res. 47, 3811–3827. 10.1093/nar/gkz120 30799503 PMC6486540

[B5] Alhammad Y. M. O. Fehr A. R. (2020). The Viral Macrodomain Counters Host Antiviral ADP-Ribosylation. Viruses 12, 384. 10.3390/v12040384 PMC7232374 32244383

[B6] Ambrose H. E. Willimott S. Beswick R. W. Dantzer F. De Murcia J. M. Yelamos J. (2009). Poly(ADP-ribose) Polymerase-1 (Parp-1)-Deficient Mice Demonstrate Abnormal Antibody Responses. Immunology 127, 178–186. 10.1111/j.1365-2567.2008.02921.x 18778284 PMC2691783

[B7] Andreone T. L. O’Connor M. Denenberg A. Hake P. W. Zingarelli B. (2003). Poly(ADP-Ribose) Polymerase-1 Regulates Activation of Activator Protein-1 in Murine Fibroblasts. J. Immunol. 170, 2113–2120. 10.4049/jimmunol.170.4.2113 12574383

[B8] Ariumi Y. Turelli P. Masutani M. Trono D. (2005). DNA Damage Sensors ATM, ATR, DNA-PKcs, and PARP-1 Are Dispensable for Human Immunodeficiency Virus Type 1 Integration. J. Virol. 79, 2973–2978. 10.1128/jvi.79.5.2973-2978.2005 15709017 PMC548471

[B9] Atasheva S. Akhrymuk M. Frolova E. I. Frolov I. (2012). New PARP Gene with an Anti-alphavirus Function. J. Virol. 86, 8147–8160. 10.1128/jvi.00733-12 22623789 PMC3421642

[B10] Atasheva S. Frolova E. I. Frolov I. (2014). Interferon-Stimulated Poly(ADP-Ribose) Polymerases Are Potent Inhibitors of Cellular Translation and Virus Replication. J. Virol. 88, 2116–2130. 10.1128/jvi.03443-13 24335297 PMC3911523

[B11] Audrito V. Managò A. Gaudino F. Sorci L. Messana V. G. Raffaelli N. (2019). NAD-biosynthetic and Consuming Enzymes as Central Players of Metabolic Regulation of Innate and Adaptive Immune Responses in Cancer. Front. Immunol. 10. 10.3389/fimmu.2019.01720 PMC6671870 31402913

[B12] Ba X. Gupta S. Davidson M. Garg N. J. (2010). Trypanosoma Cruzi Induces the Reactive Oxygen Species-PARP-1-RelA Pathway for Up-Regulation of Cytokine Expression in Cardiomyocytes. J. Biol. Chem. 285, 11596–11606. 10.1074/jbc.M109.076984 20145242 PMC2857037

[B13] Bai P. (2015). Biology of Poly(ADP-Ribose) Polymerases: The Factotums of Cell Maintenance. Mol. Cel. 58, 947–958. 10.1016/j.molcel.2015.01.034 26091343

[B14] Bamunuarachchi G. Yang X. Huang C. Liang Y. Guo Y. Liu L. (2021). MicroRNA ‐206 Inhibits Influenza A Virus Replication by Targeting Tankyrase 2. Cell Microbiol. 23. 10.1111/cmi.13281 PMC8279255 33099847

[B15] Berger F. Lau C. Dahlmann M. Ziegler M. (2005). Subcellular Compartmentation and Differential Catalytic Properties of the Three Human Nicotinamide Mononucleotide Adenylyltransferase Isoforms. J. Biol. Chem. 280, 36334–36341. 10.1074/jbc.M508660200 16118205

[B16] Berger N. A. Sims J. L. Catino D. M. Berger S. J. (2020). Poly(ADP-ribose) Polymerase Mediates the Suicide Response to Massive DNA Damage: Studies in normal and DNA-Repair Defective Cells. Princess Takamatsu Symp., 219–226. 10.1201/9781003079491-24 6317637

[B17] Bhalla S. Gordon L. I. (2016). Functional Characterization of NAD Dependent De-acetylases SIRT1 and SIRT2 in B-Cell Chronic Lymphocytic Leukemia (CLL). Cancer Biol. Ther. 17, 300–309. 10.1080/15384047.2016.1139246 26794150 PMC4847985

[B18] Bian C. Zhang C. Luo T. Vyas A. Chen S.-H. Liu C. (2019). NADP+ Is an Endogenous PARP Inhibitor in DNA Damage Response and Tumor Suppression. Nat. Commun. 10, 1–14. 10.1038/s41467-019-08530-5 30741937 PMC6370829

[B19] Bieganowski P. Brenner C. (2004). Discoveries of Nicotinamide Riboside as a Nutrient and Conserved NRK Genes Establish a Preiss-Handler Independent Route to NAD+ in Fungi and Humans. Cell 117, 495–502. 10.1016/S0092-8674(04)00416-7 15137942

[B20] Booiman T. Wit F. W. Girigorie A. F. Maurer I. De Francesco D. Sabin C. A. (2017). Terminal Differentiation of T Cells Is Strongly Associated with CMV Infection and Increased in HIV-Positive Individuals on ART and Lifestyle Matched Controls. PLoS One 12, e0183357. 10.1371/journal.pone.0183357 28806406 PMC5555623

[B21] Boulle N. Jones E. M. Auguste P. Baird A. (1995). Adenosine Diphosphate Ribosylation of Fibroblast Growth Factor-2. Mol. Endocrinol. 9, 767–775. 10.1210/MEND.9.6.8592522 8592522

[B22] Bradshaw P. (2019). Cytoplasmic and Mitochondrial NADPH-Coupled Redox Systems in the Regulation of Aging. Nutrients 11, 504. 10.3390/nu11030504 PMC6471790 30818813

[B23] Bruzzone S. Fruscione F. Morando S. Ferrando T. Poggi A. Garuti A. (2009). Catastrophic NAD+ Depletion in Activated T Lymphocytes through Nampt Inhibition Reduces Demyelination and Disability in EAE. PLoS One 4, e7897. 10.1371/journal.pone.0007897 19936064 PMC2774509

[B24] Bruzzone S. Moreschi I. Guida L. Usai C. Zocchi E. De Flora A. (2006). Extracellular NAD+ Regulates Intracellular Calcium Levels and Induces Activation of Human Granulocytes. Biochem. J. 393, 697–704. 10.1042/BJ20051302 16225456 PMC1360722

[B25] Budayeva H. G. Rowland E. A. Cristea I. M. (2016). Intricate Roles of Mammalian Sirtuins in Defense against Viral Pathogens. J. Virol. 90, 5–8. 10.1128/jvi.03220-14 26491165 PMC4702534

[B26] Bueno M. T. D. Reyes D. Valdes L. Saheba A. Urias E. Mendoza C. (2013). Poly(ADP-Ribose) Polymerase 1 Promotes Transcriptional Repression of Integrated Retroviruses. J. Virol. 87, 2496–2507. 10.1128/jvi.01668-12 23255787 PMC3571415

[B27] Camacho-Pereira J. Tarragó M. G. Chini C. C. S. Nin V. Escande C. Warner G. M. (2016). CD38 Dictates Age-Related NAD Decline and Mitochondrial Dysfunction through an SIRT3-dependent Mechanism. Cel. Metab. 23, 1127–1139. 10.1016/j.cmet.2016.05.006 PMC4911708 27304511

[B28] Cambronne X. A. Stewart M. L. Kim D. Jones-Brunette A. M. Morgan R. K. Farrens D. L. (2016). Biosensor Reveals Multiple Sources for Mitochondrial NAD+. Science 352, 1474–1477. 10.1126/science.aad5168 27313049 PMC6530784

[B29] Cameron A. M. Castoldi A. Sanin D. E. Flachsmann L. J. Field C. S. Puleston D. J. (2019). Inflammatory Macrophage Dependence on NAD+ Salvage Is a Consequence of Reactive Oxygen Species-Mediated DNA Damage. Nat. Immunol. 20, 420–432. 10.1038/s41590-019-0336-y 30858618 PMC12842115

[B30] Cantó C. Houtkooper R. H. Pirinen E. Youn D. Y. Oosterveer M. H. Cen Y. (2012). The NAD+ Precursor Nicotinamide Riboside Enhances Oxidative Metabolism and Protects against High-Fat Diet-Induced Obesity. Cel. Metab. 15, 838–847. 10.1016/j.cmet.2012.04.022 PMC3616313 22682224

[B31] Caprara G. Prosperini E. Piccolo V. Sigismondo G. Melacarne A. Cuomo A. (2018). PARP14 Controls the Nuclear Accumulation of a Subset of Type I IFN-Inducible Proteins. J.I. 200, 2439–2454. 10.4049/jimmunol.1701117 29500242

[B32] Cardoso F. Castro F. Moreira-Teixeira L. Sousa J. Torrado E. Silvestre R. (2015). Myeloid Sirtuin 2 Expression Does Not Impact Long-Term *Mycobacterium tuberculosis* Control. PLoS One 10, e0131904. 10.1371/journal.pone.0131904 26135889 PMC4489762

[B33] Carty M. Goodbody R. Schröder M. Stack J. Moynagh P. N. Bowie A. G. (2006). The Human Adaptor SARM Negatively Regulates Adaptor Protein TRIF-dependent Toll-like Receptor Signaling. Nat. Immunol. 7, 1074–1081. 10.1038/ni1382 16964262

[B34] Castro V. Bertrand L. Luethen M. Dabrowski S. Lombardi J. Morgan L. (2016). Occludin Controls HIV Transcription in Brain Pericytes via Regulation of SIRT‐1 Activation. FASEB j. 30, 1234–1246. 10.1096/fj.15-277673 26601824 PMC4750406

[B35] Chambon P. Weill J. D. Doly J. Strosser M. T. Mandel P. (1966). On the Formation of a Novel Adenylic Compound by Enzymatic Extracts of Liver Nuclei. Biochem. Biophys. Res. Commun. 25, 567–704. 10.1016/0006-291X(66)90502-X

[B36] Chambon P. Weill J. D. Mandel P. (1963). Nicotinamide Mononucleotide Activation of a New DNA-dependent Polyadenylic Acid Synthesizing Nuclear Enzyme. Biochem. Biophys. Res. Commun. 11, 39–43. 10.1016/0006-291X(63)90024-X 14019961

[B37] Chemudupati M. Kenney A. D. Bonifati S. Zani A. McMichael T. M. Wu L. (2019). From APOBEC to ZAP: Diverse Mechanisms Used by Cellular Restriction Factors to Inhibit Virus Infections. Biochim. Biophys. Acta (Bba) - Mol. Cel Res. 1866, 382–394. 10.1016/j.bbamcr.2018.09.012 PMC6334645 30290238

[B38] Chen L. Nye D. M. Stone M. C. Weiner A. T. Gheres K. W. Xiong X. (2016). Mitochondria and Caspases Tune Nmnat-Mediated Stabilization to Promote Axon Regeneration. Plos Genet. 12, e1006503. 10.1371/journal.pgen.1006503 27923046 PMC5173288

[B39] Chen Y.-H. Sasaki Y. DiAntonio A. Milbrandt J. (2021). SARM1 Is Required in Human Derived Sensory Neurons for Injury-Induced and Neurotoxic Axon Degeneration. Exp. Neurol. 339, 113636. 10.1016/j.expneurol.2021.113636 33548217 PMC8171232

[B40] Cheng C. Y. Gutierrez N. M. Marzuki M. B. Lu X. Foreman T. W. Paleja B. (2017). Host Sirtuin 1 Regulates Mycobacterial Immunopathogenesis and Represents a Therapeutic Target against Tuberculosis. Sci. Immunol. 2, eaaj1789. 10.1126/sciimmunol.aaj1789 28707004 PMC5505666

[B41] Cheong W.-C. Park J.-H. Kang H.-R. Song M. J. (2015). Downregulation of Poly(ADP-Ribose) Polymerase 1 by a Viral Processivity Factor Facilitates Lytic Replication of Gammaherpesvirus. J. Virol. 89, 9676–9682. 10.1128/jvi.00559-15 26157130 PMC4542354

[B42] Chini C. C. S. Peclat T. R. Warner G. M. Kashyap S. Espindola-Netto J. M. de Oliveira G. C. (2020). CD38 Ecto-Enzyme in Immune Cells Is Induced during Aging and Regulates NAD+ and NMN Levels. Nat. Metab. 2, 1284–1304. 10.1038/s42255-020-00298-z 33199925 PMC8752031

[B43] Cho S. H. Goenka S. Henttinen T. Gudapati P. Reinikainen A. Eischen C. M. (2009). PARP-14, a Member of the B Aggressive Lymphoma Family, Transduces Survival Signals in Primary B Cells. Blood 113, 2416–2425. 10.1182/blood-2008-03-144121 19147789 PMC2656269

[B44] Choe C.-u. Lardong K. Gelderblom M. Ludewig P. Leypoldt F. Koch-Nolte F. (2011). CD38 Exacerbates Focal Cytokine Production, Postischemic Inflammation and Brain Injury after Focal Cerebral Ischemia. PLoS One 6, e19046. 10.1371/journal.pone.0019046 21625615 PMC3097994

[B45] Ciarlo E. Heinonen T. Lugrin J. Acha-Orbea H. Le Roy D. Auwerx J. (2017). Sirtuin 3 Deficiency Does Not Alter Host Defenses against Bacterial and Fungal Infections. Sci. Rep. 7, 1–10. 10.1038/s41598-017-04263-x 28634345 PMC5478639

[B46] Clapper D. L. Walseth T. F. Dargie P. J. Lee H. C. (1987). Pyridine Nucleotide Metabolites Stimulate Calcium Release from Sea Urchin Egg Microsomes Desensitized to Inositol Trisphosphate. J. Biol. Chem. 262, 9561–9568. 10.1016/s0021-9258(18)47970-7 3496336

[B47] Clouser C. L. Chauhan J. Bess M. A. Oploo J. L. V. Zhou D. Dimick-Gray S. (2012). Anti-HIV-1 Activity of Resveratrol Derivatives and Synergistic Inhibition of HIV-1 by the Combination of Resveratrol and Decitabine. Bioorg. Med. Chem. Lett. 22, 6642–6646. 10.1016/j.bmcl.2012.08.108 23010273 PMC3482103

[B48] Cortés-Garcia J. D. López-López C. Cortez-Espinosa N. García-Hernández M. H. Guzmán-Flores J. M. Layseca-Espinosa E. (2016). Evaluation of the Expression and Function of the P2X7 Receptor and ART1 in Human Regulatory T-Cell Subsets. Immunobiology 221, 84–93. 10.1016/J.IMBIO.2015.07.018 26307000

[B49] Covarrubias A. J. Kale A. Perrone R. Lopez-Dominguez J. A. Pisco A. O. Kasler H. G. (2021). Author Correction: Senescent Cells Promote Tissue NAD+ Decline during Ageing via the Activation of CD38+ Macrophages. Nat. Metab. 3, 120–121. 10.1038/s42255-020-00328-w 33303985

[B50] Curtin N. Bányai K. Thaventhiran J. Le Quesne J. Helyes Z. Bai P. (2020). Repositioning PARP Inhibitors for SARS‐CoV‐2 infection(COVID‐19); a New Multi‐pronged Therapy for Acute Respiratory Distress Syndrome? Br. J. Pharmacol. 177, 3635–3645. 10.1111/bph.15137 32441764 PMC7280733

[B51] Daugherty M. D. Young J. M. Kerns J. A. Malik H. S. (2014). Rapid Evolution of PARP Genes Suggests a Broad Role for ADP-Ribosylation in Host-Virus Conflicts. Plos Genet. 10, e1004403. 10.1371/journal.pgen.1004403 24875882 PMC4038475

[B52] David K. K. Andrabi S. A. Dawson T. M. Dawson V. L. (2009). Parthanatos, A Messenger of Death. Front. Biosci. 14, 1116. 10.2741/3297 PMC4450718 19273119

[B53] Davidovic L. Vodenicharov M. Affar E. B. Poirier G. G. (2001). Importance of Poly(adp-Ribose) Glycohydrolase in the Control of Poly(adp-Ribose) Metabolism. Exp. Cel Res. 268, 7–13. 10.1006/excr.2001.5263 11461113

[B54] Davila A. Liu L. Chellappa K. Redpath P. Nakamaru-Ogiso E. Paolella L. M. (2018). Nicotinamide Adenine Dinucleotide Is Transported into Mammalian Mitochondria. Elife 7. 10.7554/eLife.33246 PMC6013257 29893687

[B55] Deng Z. Atanasiu C. Zhao K. Marmorstein R. Sbodio J. I. Chi N.-W. (2005). Inhibition of Epstein-Barr Virus OriP Function by Tankyrase, a Telomere-Associated Poly-ADP Ribose Polymerase that Binds and Modifies EBNA1. J. Virol. 79, 4640–4650. 10.1128/jvi.79.8.4640-4650.2005 15795250 PMC1069541

[B56] Di Girolamo M. Fabrizio G. (2019). Overview of the Mammalian ADP-Ribosyl-Transferases Clostridia Toxin-like (ARTCs) Family. Biochem. Pharmacol. 167, 86–96. 10.1016/J.BCP.2019.07.004 31283932

[B57] Diray-Arce J. Conti M. G. Petrova B. Kanarek N. Angelidou A. Levy O. (2020). Integrative Metabolomics to Identify Molecular Signatures of Responses to Vaccines and Infections. Metabolites 10, 492. 10.3390/metabo10120492 PMC7760881 33266347

[B58] Doly J. Petek F. (1966). Etude de la structure d’un compose “poly(ADP-ribose” synthetise par des extraits nucleaires de foie de poulet. C. R. Hebd. Seances Acad. Sci. Ser. D Sci. Nat. 263, 1341–1344.

[B59] Eisenreich W. Rudel T. Heesemann J. Goebel W. (2019). How Viral and Intracellular Bacterial Pathogens Reprogram the Metabolism of Host Cells to Allow Their Intracellular Replication. Front. Cell. Infect. Microbiol. 9, 42. 10.3389/fcimb.2019.00042 30886834 PMC6409310

[B60] Elesela S. Morris S. B. Narayanan S. Kumar S. Lombard D. B. Lukacs N. W. (2020). Sirtuin 1 Regulates Mitochondrial Function and Immune Homeostasis in Respiratory Syncytial Virus Infected Dendritic Cells. PLOS Pathog. 16, e1008319. 10.1371/journal.ppat.1008319 32106265 PMC7046194

[B61] Elhassan Y. S. Kluckova K. Fletcher R. S. Schmidt M. S. Garten A. Doig C. L. (2019). Nicotinamide Riboside Augments the Aged Human Skeletal Muscle NAD+ Metabolome and Induces Transcriptomic and Anti-inflammatory Signatures. Cel Rep. 28, 1717–1728. 10.1016/j.celrep.2019.07.043 PMC6702140 31412242

[B62] Escande C. Nin V. Price N. L. Capellini V. Gomes A. P. Barbosa M. T. (2013). Flavonoid Apigenin Is an Inhibitor of the NAD+ase CD38: Implications for Cellular NAD+ Metabolism, Protein Acetylation, and Treatment of Metabolic Syndrome. Diabetes 62, 1084–1093. 10.2337/db12-1139 23172919 PMC3609577

[B63] Eskandarian H. A. Impens F. Nahori M.-A. Soubigou G. Coppée J.-Y. Cossart P. (2013). A Role for SIRT2-dependent Histone H3K18 Deacetylation in Bacterial Infection. Science 341, 1238858. 10.1126/science.1238858 23908241

[B64] Essuman K. Summers D. W. Sasaki Y. Mao X. DiAntonio A. Milbrandt J. (2017). The SARM1 Toll/Interleukin-1 Receptor Domain Possesses Intrinsic NAD + Cleavage Activity that Promotes Pathological Axonal Degeneration. Neuron 93, 1334–1343. 10.1016/j.neuron.2017.02.022 28334607 PMC6284238

[B65] Evans C. Bogan K. L. Song P. Burant C. F. Kennedy R. T. Brenner C. (2010). NAD+ Metabolite Levels as a Function of Vitamins and Calorie Restriction: Evidence for Different Mechanisms of Longevity. BMC Chem. Biol. 10, 2. 10.1186/1472-6769-10-2 20175898 PMC2834649

[B66] Fabrizio G. Di Paola S. Stilla A. Giannotta M. Ruggiero C. Menzel S. (2015). ARTC1-mediated ADP-Ribosylation of GRP78/BiP: A New Player in Endoplasmic-Reticulum Stress Responses. Cell. Mol. Life Sci. 72, 1209–1225. 10.1007/s00018-014-1745-6 25292337 PMC11113179

[B67] Fletcher R. S. Ratajczak J. Doig C. L. Oakey L. A. Callingham R. Da Silva Xavier G. (2017). Nicotinamide Riboside Kinases Display Redundancy in Mediating Nicotinamide Mononucleotide and Nicotinamide Riboside Metabolism in Skeletal Muscle Cells. Mol. Metab. 6, 819–832. 10.1016/j.molmet.2017.05.011 28752046 PMC5518663

[B68] Fujimura S. Hasegawa S. Shimizu Y. Sugimura T. (1967). Polymerization of the Adenosine 5′-Diphosphate-Ribose Moiety of Nicotinamide-Adenine Dinucleotide by Nuclear Enzyme. BBA Sect. Nucleic Acids Protein Synth. 145, 247–259. 10.1016/0005-2787(67)90043-3 4294274

[B69] Funaro A. De Monte L. B. Dianzani U. Forni M. Malavasi F. (1993). Human CD38 Is Associated to Distinct Molecules Which Mediate Transmembrane Signaling in Different Lineages. Eur. J. Immunol. 23, 2407–2411. 10.1002/eji.1830231005 8405040

[B70] Galindo-Campos M. A. Bedora-Faure M. Farrés J. Lescale C. Moreno-Lama L. Martínez C. (2019). Coordinated Signals from the DNA Repair Enzymes PARP-1 and PARP-2 Promotes B-Cell Development and Function. Cell Death Differ 26, 2667–2681. 10.1038/s41418-019-0326-5 30996287 PMC6861126

[B71] Gan H. Shen T. Chupp D. P. Taylor J. R. Sanchez H. N. Li X. (2020). B Cell Sirt1 Deacetylates Histone and Non-histone Proteins for Epigenetic Modulation of AID Expression and the Antibody Response. Sci. Adv. 6, eaay2793. 10.1126/sciadv.aay2793 32270032 PMC7112761

[B72] Geisler S. Huang S. X. Strickland A. Doan R. A. Summers D. W. Mao X. (2019). Gene Therapy Targeting SARM1 Blocks Pathological Axon Degeneration in Mice. J. Exp. Med. 216, 294–303. 10.1084/jem.20181040 30642945 PMC6363435

[B73] Gerdts J. Brace E. J. Sasaki Y. DiAntonio A. Milbrandt J. (2015). SARM1 Activation Triggers Axon Degeneration Locally via NAD+ Destruction. Science 348, 453–457. 10.1126/science.1258366 25908823 PMC4513950

[B74] Gibbs-Seymour I. Fontana P. Rack J. G. M. Ahel I. (2016). HPF1/C4orf27 Is a PARP-1-Interacting Protein that Regulates PARP-1 ADP-Ribosylation Activity. Mol. Cel 62, 432–442. 10.1016/j.molcel.2016.03.008 PMC4858568 27067600

[B75] Giroud-Gerbetant J. Joffraud M. Giner M. P. Cercillieux A. Bartova S. Makarov M. V. (2019). A Reduced Form of Nicotinamide Riboside Defines a New Path for NAD+ Biosynthesis and Acts as an Orally Bioavailable NAD+ Precursor. Mol. Metab. 30, 192–202. 10.1016/j.molmet.2019.09.013 31767171 PMC6807296

[B76] Glaría E. Valledor A. F. (2020). Roles of CD38 in the Immune Response to Infection. Cells 9, 228. 10.3390/cells9010228 PMC7017097 31963337

[B77] Glowacki G. Braren R. Firner K. Nissen M. Kühl M. Reche P. (2002). The Family of Toxin-Related Ecto-ADP-Ribosyltransferases in Humans and the Mouse. Protein Sci. 11, 1657–1670. 10.1110/PS.0200602 12070318 PMC2373659

[B78] Goody M. F. Henry C. A. (2018). A Need for NAD+ in Muscle Development, Homeostasis, and Aging. Skeletal Muscle 8. 10.1186/s13395-018-0154-1 PMC5840929 29514713

[B79] Gossmann T. I. Ziegler M. (2014). Sequence Divergence and Diversity Suggests Ongoing Functional Diversification of Vertebrate NAD Metabolism. DNA Repair 23, 39–48. 10.1016/j.dnarep.2014.07.005 25084685 PMC4248024

[B80] Grady S. L. Hwang J. Vastag L. Rabinowitz J. D. Shenk T. (2012). Herpes Simplex Virus 1 Infection Activates Poly(ADP-Ribose) Polymerase and Triggers the Degradation of Poly(ADP-Ribose) Glycohydrolase. J. Virol. 86, 8259–8268. 10.1128/jvi.00495-12 22623791 PMC3421676

[B81] Grunewald M. E. Chen Y. Kuny C. Maejima T. Lease R. Ferraris D. (2019a). The Coronavirus Macrodomain Is Required to Prevent PARP-Mediated Inhibition of Virus Replication and Enhancement of IFN Expression. Plos Pathog. 15, e1007756. 10.1371/journal.ppat.1007756 31095648 PMC6521996

[B82] Grunewald M. E. Shaban M. G. Mackin S. R. Fehr A. R. Perlman S. (2020b). Murine Coronavirus Infection Activates the Aryl Hydrocarbon Receptor in an Indoleamine 2,3-dioxygenase-independent Manner, Contributing to Cytokine Modulation and Proviral TCDD-Inducible-PARP Expression. J. Virol. 94. 10.1128/jvi.01743-19 PMC7000979 31694960

[B83] Guedes A. G. Dileepan M. Jude J. A. Deshpande D. A. Walseth T. F. Kannan M. S. (2020). Role of CD38/cADPR Signaling in Obstructive Pulmonary Diseases. Curr. Opin. Pharmacol. 51, 29–33. 10.1016/j.coph.2020.04.007 32480246 PMC7529733

[B84] Guo T. Zuo Y. Qian L. Liu J. Yuan Y. Xu K. (2019). ADP-ribosyltransferase PARP11 Modulates the Interferon Antiviral Response by Mono-ADP-Ribosylating the Ubiquitin E3 Ligase β-TrCP. Nat. Microbiol. 4, 1872–1884. 10.1038/s41564-019-0428-3 30988430

[B85] Guo X. Ma J. Sun J. Gao G. (2007). The Zinc-finger Antiviral Protein Recruits the RNA Processing Exosome to Degrade the Target mRNA. Proc. Natl. Acad. Sci. 104, 151–156. 10.1073/pnas.0607063104 17185417 PMC1765426

[B86] Gutierrez D. A. Valdes L. Serguera C. Llano M. (2016). Poly(ADP-ribose) Polymerase-1 Silences Retroviruses Independently of Viral DNA Integration or Heterochromatin Formation. J. Gen. Virol. 97, 1686–1692. 10.1099/jgv.0.000466 27028089 PMC7011754

[B87] Ha H. C. Juluri K. Zhou Y. Leung S. Hermankova M. Snyder S. H. (2001). Poly(ADP-ribose) Polymerase-1 Is Required for Efficient HIV-1 Integration. Proc. Natl. Acad. Sci. 98, 3364–3368. 10.1073/pnas.051633498 11248084 PMC30659

[B88] Han J. Chen C. Wang C. Qin N. Huang M. Ma Z. (2020). Transcriptome‐wide Association Study for Persistent Hepatitis B Virus Infection and Related Hepatocellular Carcinoma. Liver Int. 40, 2117–2127. 10.1111/liv.14577 32574393

[B89] Harden A. Young W. (1906). The Alcoholic Ferment of Yeast-Juice. Proc. R. Soc. Lond. B. 77, 405–420. 10.1098/rspb.1906.0029

[B90] Hassa P. O. Hottiger M. O. (2008). The Diverse Biological Roles of Mammalian PARPs, a Small but Powerful Family of Poly-ADP-Ribose Polymerases. Front. Biosci. 13, 3046. 10.2741/2909 17981777

[B91] Hauser S. Adelmant G. Sarraf P. Wright H. M. Mueller E. Spiegelman B. M. (2000). Degradation of the Peroxisome Proliferator-Activated Receptor γ Is Linked to Ligand-dependent Activation. J. Biol. Chem. 275, 18527–18533. 10.1074/jbc.M001297200 10748014

[B92] He Y. Yang G. Sun L. Gao H. Yao F. Jin Z. (2021). SIRT6 Inhibits Inflammatory Response through Regulation of NRF2 in Vascular Endothelial Cells. Int. Immunopharmacology 99, 107926. 10.1016/j.intimp.2021.107926 34233231

[B93] Héberlé E. Amé J.-C. Illuzzi G. Dantzer F. Schreiber V. (2015). Discovery of the Parp Superfamily and Focus on the Lesser Exhibited but Not Lesser Talented Members. Cancer Drug Discov. Dev. 83, 15–46. 10.1007/978-3-319-14151-0_2

[B94] Heer C. D. Sanderson D. J. Voth L. S. Alhammad Y. M. O. Schmidt M. S. Trammell S. A. J. (2020). Coronavirus Infection and PARP Expression Dysregulate the NAD Metabolome: an Actionable Component of Innate Immunity. bioRxiv. 10.1101/2020.04.17.047480 PMC7834058 33051211

[B95] Heinonen T. Ciarlo E. Rigoni E. Regina J. Le Roy D. Roger T. (2019). Dual Deletion of the Sirtuins SIRT2 and SIRT3 Impacts on Metabolism and Inflammatory Responses of Macrophages and Protects from Endotoxemia. Front. Immunol. 10, 2713. 10.3389/fimmu.2019.02713 31849939 PMC6901967

[B96] Heinonen T. Ciarlo E. Théroude C. Pelekanou A. Herderschee J. Le Roy D. (2018). Sirtuin 5 Deficiency Does Not Compromise Innate Immune Responses to Bacterial Infections. Front. Immunol. 9. 10.3389/fimmu.2018.02675 PMC6255879 30515162

[B97] Hernández-Campo P. M. Almeida J. Sánchez M. L. Malvezzi M. Orfao A. (2006). Normal Patterns of Expression of Glycosylphosphatidylinositol-Anchored Proteins on Different Subsets of Peripheral Blood Cells: A Frame of Reference for the Diagnosis of Paroxysmal Nocturnal Hemoglobinuria. Cytometry 70B, 71–81. 10.1002/cyto.b.20087 16493662

[B98] Hinz M. Stilmann M. Arslan S. Ç. Khanna K. K. Dittmar G. Scheidereit C. (2010). A Cytoplasmic ATM-TRAF6-cIAP1 Module Links Nuclear DNA Damage Signaling to Ubiquitin-Mediated NF-Κb Activation. Mol. Cel. 40, 63–74. 10.1016/j.molcel.2010.09.008 20932475

[B99] Hong S. Brass A. Seman M. Haag F. Koch-Nolte F. Dubyak G. R. (2009). Basal and Inducible Expression of the Thiol-Sensitive ART2.1 Ecto-ADP-Ribosyltransferase in Myeloid and Lymphoid Leukocytes. Purinergic Signal. 5, 369–383. 10.1007/S11302-009-9162-2 19404775 PMC2717319

[B100] Hopp A.-K. Teloni F. Bisceglie L. Gondrand C. Raith F. Nowak K. (2021). Mitochondrial NAD+ Controls Nuclear ARTD1-Induced ADP-Ribosylation. Mol. Cel. 81, 340–354. 10.1016/j.molcel.2020.12.034 PMC7837215 33450210

[B101] Hottiger M. O. Hassa P. O. Lüscher B. Schüler H. Koch-Nolte F. (2010). Toward a Unified Nomenclature for Mammalian ADP-Ribosyltransferases. Trends Biochem. Sci. 35, 208–219. 10.1016/J.TIBS.2009.12.003 20106667

[B102] Hou Y.-J. Banerjee R. Thomas B. Nathan C. García-Sastre A. Ding A. (2013). SARM Is Required for Neuronal Injury and Cytokine Production in Response to Central Nervous System Viral Infection. J.I. 191, 875–883. 10.4049/jimmunol.1300374 PMC3710687 23749635

[B103] Hsieh C.-L. Huang H.-M. Hsieh S.-Y. Zheng P.-X. Lin Y.-S. Chiang-Ni C. (2018). NAD-glycohydrolase Depletes Intracellular NAD+ and Inhibits Acidification of Autophagosomes to Enhance Multiplication of Group A Streptococcus in Endothelial Cells. Front. Microbiol. 9. 10.3389/fmicb.2018.01733 PMC6085451 30123194

[B104] Hussain A. M. M. Lee H. C. Chang C. F. (1998). Functional Expression of Secreted Mouse BST-1 in Yeast. Protein Expr. Purif. 12, 133–137. 10.1006/prep.1997.0811 9473467

[B105] Imperatore F. Maurizio J. Vargas Aguilar S. Busch C. J. Favret J. Kowenz‐Leutz E. (2017). SIRT1 Regulates Macrophage Self‐renewal. EMBO J. 36, 2353–2372. 10.15252/embj.201695737 28701484 PMC5556267

[B106] Ivy J. M. Klar A. J. Hicks J. B. (1986). Cloning and Characterization of Four SIR Genes of *Saccharomyces cerevisiae* . Mol. Cell. Biol. 6, 688–702. 10.1128/mcb.6.2.688 3023863 PMC367560

[B107] Iwata H. Goettsch C. Sharma A. Ricchiuto P. Goh W. W. B. Halu A. (2016). PARP9 and PARP14 Cross-Regulate Macrophage Activation via STAT1 ADP-Ribosylation. Nat. Commun. 7, 1–19. 10.1038/ncomms12849 PMC5095532 27796300

[B108] Jacobson M. K. Amé J. C. Lin W. Coyle D. L. Jacobson E. L. (1995). Cyclic ADP-Ribose. A New Component of Calcium Signaling. Receptor 5, 43–49. 7613483

[B109] Jeng M. Y. Hull P. A. Fei M. Kwon H.-S. Tsou C.-L. Kasler H. (2018). Metabolic Reprogramming of Human CD8+ Memory T Cells through Loss of SIRT1. J. Exp. Med. 215, 51–62. 10.1084/jem.20161066 29191913 PMC5748845

[B110] Jiang C. Liu J. Guo M. Gao X. Wu X. Bai N. (2020). The Nad-dependent Deacetylase SIRT2 Regulates T Cell Differentiation Involved in Tumor Immune Response. Int. J. Biol. Sci. 16, 3075–3084. 10.7150/ijbs.49735 33061819 PMC7545715

[B111] Jones E. M. Baird A. (1997). Cell-surface ADP-Ribosylation of Fibroblast Growth Factor-2 by an Arginine-specific ADP-Ribosyltransferase. Biochem. J. 323, 173–177. 10.1042/bj3230173 9173879 PMC1218292

[B112] Kaeberlein M. McVey M. Guarente L. (1999). The SIR2/3/4 Complex and SIR2 Alone Promote Longevity in *Saccharomyces cerevisiae* by Two Different Mechanisms. Genes Develop. 13, 2570–2580. 10.1101/gad.13.19.2570 10521401 PMC317077

[B113] Kahl S. Nissen M. Girisch R. Duffy T. Leiter E. H. Haag F. (2000). Metalloprotease-Mediated Shedding of Enzymatically Active Mouse Ecto-ADP-Ribosyltransferase ART2.2 upon T Cell Activation. J. Immunol. 165, 4463–4469. 10.4049/jimmunol.165.8.4463 11035085

[B114] Kameoka M. Nukuzuma S. Itaya A. Tanaka Y. Ota K. Ikuta K. (2004). RNA Interference Directed against Poly(ADP-Ribose) Polymerase 1 Efficiently Suppresses Human Immunodeficiency Virus Type 1 Replication in Human Cells. J. Virol. 78, 8931–8934. 10.1128/jvi.78.16.8931-8934.2004 15280503 PMC479071

[B115] Kameoka M. Nukuzuma S. Itaya A. Tanaka Y. Ota K. Inada Y. (2005). Poly(ADP-ribose)polymerase-1 Is Required for Integration of the Human Immunodeficiency Virus Type 1 Genome Near Centromeric Alphoid DNA in Human and Murine Cells. Biochem. Biophysical Res. Commun. 334, 412–417. 10.1016/j.bbrc.2005.06.104 16002043

[B116] Kang B. N. Tirumurugaan K. G. Deshpande D. A. Amrani Y. Panettieri R. A. Walseth T. F. (2006). Transcriptional Regulation of CD38 Expression by Tumor Necrosis Factor‐α in Human Airway Smooth Muscle Cells: Role of NF‐κB and Sensitivity to Glucocorticoids. FASEB j. 20, 1000–1002. 10.1096/fj.05-4585fje 16571778

[B117] Kasamatsu A. Nakao M. Smith B. C. Comstock L. R. Ono T. Kato J. (2011). Hydrolysis of O-Acetyl-ADP-Ribose Isomers by ADP-Ribosylhydrolase 3. J. Biol. Chem. 286, 21110–21117. 10.1074/jbc.M111.237636 21498885 PMC3122172

[B118] Katsuyama E. Suarez-Fueyo A. Bradley S. J. Mizui M. Marin A. V. Mulki L. (2020). The CD38/NAD/SIRTUIN1/EZH2 Axis Mitigates Cytotoxic CD8 T Cell Function and Identifies Patients with SLE Prone to Infections. Cel. Rep. 30, 112–123.e4. 10.1016/j.celrep.2019.12.014 PMC7577012 31914379

[B119] Katsyuba E. Romani M. Hofer D. Auwerx J. (2020). NAD+ Homeostasis in Health and Disease. Nat. Metab. 2, 9–31. 10.1038/s42255-019-0161-5 32694684

[B120] Kauppinen A. Suuronen T. Ojala J. Kaarniranta K. Salminen A. (2013). Antagonistic Crosstalk between NF-Κb and SIRT1 in the Regulation of Inflammation and Metabolic Disorders. Cell Signal. 25, 1939–1948. 10.1016/j.cellsig.2013.06.007 23770291

[B121] Kawahara T. L. A. Michishita E. Adler A. S. Damian M. Berber E. Lin M. (2009). SIRT6 Links Histone H3 Lysine 9 Deacetylation to NF-κb-dependent Gene Expression and Organismal Life Span. Cell 136, 62–74. 10.1016/j.cell.2008.10.052 19135889 PMC2757125

[B122] Kim H. Jacobson E. Jacobson M. (1993a). Synthesis and Degradation of Cyclic ADP-Ribose by NAD Glycohydrolases. Science 261, 1330–1333. 10.1126/science.8395705 8395705

[B123] Kim U. H. Kim M. K. Kim J. S. Han M. K. Park B. H. Kim H. R. (1993b). Purification and Characterization of NAD Glycohydrolase from Rabbit Erythrocytes. Arch. Biochem. Biophys. 305, 147–152. 10.1006/ABBI.1993.1404 8393643

[B124] Kim Y. J. Lee S.-H. Jeon S. M. Silwal P. Seo J.-Y. Hanh B. T. B. (2020). Sirtuin 3 Is Essential for Host Defense against Mycobacterium Abscessus Infection through Regulation of Mitochondrial Homeostasis. Virulence 11, 1225–1239. 10.1080/21505594.2020.1809961 32835604 PMC7549921

[B125] Klar A. J. Fogel S. (1979). Activation of Mating Type Genes by Transposition in *Saccharomyces cerevisiae* . Proc. Natl. Acad. Sci. 76, 4539–4543. 10.1073/pnas.76.9.4539 388445 PMC411613

[B126] Klimova N. Long A. Kristian T. (2019). Nicotinamide Mononucleotide Alters Mitochondrial Dynamics by SIRT3‐dependent Mechanism in Male Mice. J. Neurosci. Res. 97, 975–990. 10.1002/jnr.24397 30801823 PMC6565489

[B127] Ko H. L. Ng H. J. Goh E. H. Ren E. C. (2013). Reduced ADP-Ribosylation by PARP1 Natural Polymorphism V762A and by PARP1 Inhibitors Enhance Hepatitis B Virus Replication. J. Viral Hepat. 20, 658–665. 10.1111/jvh.12100 23910651

[B128] Kong S. McBurney M. W. Fang D. (2012). Sirtuin 1 in Immune Regulation and Autoimmunity. Immunol. Cel Biol 90, 6–13. 10.1038/icb.2011.102 22105513

[B129] Kory N. uit de Bos J. van der Rijt S. van der Rijt N. Güra M. Arp N. (2020). MCART1/SLC25A51 Is Required for Mitochondrial NAD Transport. Sci. Adv. 6, eabe5310. 10.1126/sciadv.abe5310 33087354 PMC7577609

[B130] Koyuncu E. Budayeva H. G. Miteva Y. V. Ricci D. P. Silhavy T. J. Shenk T. (2014). Sirtuins Are Evolutionarily Conserved Viral Restriction Factors. MBio 5, 2249–2263. 10.1128/mBio.02249-14 PMC4271551 25516616

[B131] Kozaki T. Komano J. Kanbayashi D. Takahama M. Misawa T. Satoh T. (2017). Mitochondrial Damage Elicits a TCDD-Inducible poly(ADP-Ribose) Polymerase-Mediated Antiviral Response. Proc. Natl. Acad. Sci. USA 114, 2681–2686. 10.1073/pnas.1621508114 28213497 PMC5347618

[B132] Kwon H.-S. Brent M. M. Getachew R. Jayakumar P. Chen L.-F. Schnolzer M. (2008). Human Immunodeficiency Virus Type 1 Tat Protein Inhibits the SIRT1 Deacetylase and Induces T Cell Hyperactivation. Cell Host & Microbe 3, 158–167. 10.1016/j.chom.2008.02.002 18329615 PMC2680745

[B133] Landry J. Sutton A. Tafrov S. T. Heller R. C. Stebbins J. Pillus L. (2000). The Silencing Protein SIR2 and its Homologs Are NAD-dependent Protein Deacetylases. Proc. Natl. Acad. Sci. 97, 5807–5811. 10.1073/pnas.110148297 10811920 PMC18515

[B134] Langelier M.-F. Eisemann T. Riccio A. A. Pascal J. M. (2018). PARP Family Enzymes: Regulation and Catalysis of the poly(ADP-Ribose) Posttranslational Modification. Curr. Opin. Struct. Biol. 53, 187–198. 10.1016/j.sbi.2018.11.002 30481609 PMC6687463

[B135] Law L. M. J. Razooky B. S. Li M. M. H. You S. Jurado A. Rice C. M. (2019). ZAP's Stress Granule Localization Is Correlated with its Antiviral Activity and Induced by Virus Replication. Plos Pathog. 15, e1007798. 10.1371/journal.ppat.1007798 31116799 PMC6548403

[B136] Lee H. C. Aarhus R. Levitt D. (1994). The crystal Structure of Cyclic ADP-Ribose. Nat. Struct. Mol. Biol. 1, 143–144. 10.1038/nsb0394-143 7656029

[B137] Lee H. C. Graeff R. Walseth T. F. (1995). Cyclic ADP-Ribose and its Metabolic Enzymes. Biochimie 77, 345–355. 10.1016/0300-9084(96)88145-4 8527488

[B138] Lee W.-P. Hou M.-C. Lan K.-H. Li C.-P. Chao Y. Lin H.-C. (2016). Helicobacter Pylori-Induced Chronic Inflammation Causes Telomere Shortening of Gastric Mucosa by Promoting PARP-1-Mediated Non-homologous End Joining of DNA. Arch. Biochem. Biophys. 606, 90–98. 10.1016/j.abb.2016.07.014 27450718

[B139] Leung A. K. L. Vyas S. Rood J. E. Bhutkar A. Sharp P. A. Chang P. (2011). Poly(ADP-Ribose) Regulates Stress Responses and MicroRNA Activity in the Cytoplasm. Mol. Cel 42, 489–499. 10.1016/j.molcel.2011.04.015 PMC3898460 21596313

[B140] Leutert M. Menzel S. Braren R. Rissiek B. Hopp A.-K. Nowak K. (2018). Proteomic Characterization of the Heart and Skeletal Muscle Reveals Widespread Arginine ADP-Ribosylation by the ARTC1 Ectoenzyme. Cel Rep. 24, 1916–1929.e5. 10.1016/J.CELREP.2018.07.048 30110646

[B141] Li C. Debing Y. Jankevicius G. Neyts J. Ahel I. Coutard B. (2016). Viral Macro Domains Reverse Protein ADP-Ribosylation. J. Virol. 90, 8478–8486. 10.1128/jvi.00705-16 27440879 PMC5021415

[B142] Li L. Zhao H. Liu P. Li C. Quanquin N. Ji X. (2018a). PARP12suppresses Zika Virus Infection through PARP-dependent Degradation of NS1 and NS3 Viral Proteins. Sci. Signal. 11, eaas9332. 10.1126/scisignal.aas9332 29921658 PMC6434931

[B143] Li M. M. H. Aguilar E. G. Michailidis E. Pabon J. Park P. Wu X. (2019). Characterization of Novel Splice Variants of Zinc Finger Antiviral Protein (ZAP). J. Virol. 93. 10.1128/jvi.00715-19 PMC6714797 31118263

[B144] Li P. Jin Y. Qi F. Wu F. Luo S. Cheng Y. (2018b). SIRT6 Acts as a Negative Regulator in Dengue Virus-Induced Inflammatory Response by Targeting the DNA Binding Domain of NF-Κb P65. Front. Cell. Infect. Microbiol. 8, 113. 10.3389/fcimb.2018.00113 29686974 PMC5900784

[B145] Li Q. He M. Zhou F. Ye F. Gao S.-J. (2014). Activation of Kaposi's Sarcoma-Associated Herpesvirus (KSHV) by Inhibitors of Class III Histone Deacetylases: Identification of Sirtuin 1 as a Regulator of the KSHV Life Cycle. J. Virol. 88, 6355–6367. 10.1128/jvi.00219-14 24672028 PMC4093851

[B146] Li Z. Yamauchi Y. Kamakura M. Murayama T. Goshima F. Kimura H. (2012). Herpes Simplex Virus Requires Poly(ADP-Ribose) Polymerase Activity for Efficient Replication and Induces Extracellular Signal-Related Kinase-dependent Phosphorylation and ICP0-dependent Nuclear Localization of Tankyrase 1. J. Virol. 86, 492–503. 10.1128/jvi.05897-11 22013039 PMC3255871

[B147] Lin Y.-T. Chiweshe S. McCormick D. Raper A. Wickenhagen A. DeFillipis V. (2020). Human Cytomegalovirus Evades ZAP Detection by Suppressing CpG Dinucleotides in the Major Immediate Early 1 Gene. PLOS Pathog. 16, e1008844. 10.1371/journal.ppat.1008844 32886716 PMC7498042

[B148] Lischke T. Heesch K. Schumacher V. Schneider M. Haag F. Koch-Nolte F. (2013). CD38 Controls the Innate Immune Response against listeria Monocytogenes. Infect. Immun. 81, 4091–4099. 10.1128/IAI.00340-13 23980105 PMC3811837

[B149] Liu H.-w. Smith C. B. Schmidt M. S. Cambronne X. A. Cohen M. S. Migaud M. E. (2018b). Pharmacological Bypass of NAD+ Salvage Pathway Protects Neurons from Chemotherapy-Induced Degeneration. Proc. Natl. Acad. Sci. USA 115, 10654–10659. 10.1073/PNAS.1809392115 30257945 PMC6196523

[B150] Liu L. Su X. Quinn W. J. Hui S. Krukenberg K. Frederick D. W. (2018a). Quantitative Analysis of NAD Synthesis-Breakdown Fluxes. Cel Metab. 27, 1067–1080.e5. 10.1016/j.cmet.2018.03.018 PMC5932087 29685734

[B151] Liu Z.-X. Yu Y. Dennert G. (1999). A Cell Surface ADP-Ribosyltransferase Modulates T Cell Receptor Association and Signaling. J. Biol. Chem. 274, 17399–17401. 10.1074/JBC.274.25.17399 10364166

[B152] Luo L. Lucas R. M. Liu L. Stow J. L. (2019). Signalling, Sorting and Scaffolding Adaptors for Toll-like Receptors. J. Cel. Sci. 133. 10.1242/jcs.239194 31889021

[B153] Luongo T. S. Eller J. M. Lu M.-J. Niere M. Raith F. Perry C. (2020). SLC25A51 Is a Mammalian Mitochondrial NAD+ Transporter. Nature 588, 174–179. 10.1038/s41586-020-2741-7 32906142 PMC7718333

[B154] Lupey-Green L. N. Moquin S. A. Martin K. A. McDevitt S. M. Hulse M. Caruso L. B. (2017). PARP1 Restricts Epstein Barr Virus Lytic Reactivation by Binding the BZLF1 Promoter. Virology 507, 220–230. 10.1016/j.virol.2017.04.006 28456021 PMC5521201

[B155] Ma S. Mao Q. Chen W. Zhao M. Wu K. Song D. (2019). Serum Lipidomics Analysis of Classical Swine Fever Virus Infection in Piglets and Emerging Role of Free Fatty Acids in Virus Replication *In Vitro* . Front. Cell. Infect. Microbiol. 9, 410. 10.3389/fcimb.2019.00410 31850242 PMC6901794

[B156] Mahmoud L. Al-Saif M. Amer H. M. Sheikh M. Almajhdi F. N. Khabar K. S. A. (2011). Green Fluorescent Protein Reporter System with Transcriptional Sequence Heterogeneity for Monitoring the Interferon Response. J. Virol. 85, 9268–9275. 10.1128/jvi.00772-11 21752918 PMC3165742

[B157] Marazzi I. Ho J. S. Y. Kim J. Manicassamy B. Dewell S. Albrecht R. A. (2012). Suppression of the Antiviral Response by an Influenza Histone Mimic. Nature 483, 428–433. 10.1038/nature10892 22419161 PMC3598589

[B158] Matalonga J. Glaria E. Bresque M. Escande C. Carbó J. M. Kiefer K. (2017). The Nuclear Receptor LXR Limits Bacterial Infection of Host Macrophages through a Mechanism that Impacts Cellular NAD Metabolism. Cel Rep. 18, 1241–1255. 10.1016/j.celrep.2017.01.007 28147278

[B159] Maurya S. P. Das B. K. Singh R. Tyagi S. (2019). Effect of Withania Somnifer on CD38 Expression on CD8+ T Lymphocytes Among Patients of HIV Infection. Clin. Immunol. 203, 122–124. 10.1016/j.clim.2019.04.003 31004791

[B160] Mehrotra P. Riley J. P. Patel R. Li F. Voss L. e. Goenka S. (2011). PARP-14 Functions as a Transcriptional Switch for Stat6-dependent Gene Activation. J. Biol. Chem. 286, 1767–1776. 10.1074/jbc.M110.157768 21081493 PMC3023471

[B161] Michos A. Gryllos I. Håkansson A. Srivastava A. Kokkotou E. Wessels M. R. (2006). Enhancement of Streptolysin O Activity and Intrinsic Cytotoxic Effects of the Group A Streptococcal Toxin, NAD-Glycohydrolase. J. Biol. Chem. 281, 8216–8223. 10.1074/jbc.M511674200 16431917

[B162] Migaud M. Gandotra S. Chand H. S. Gillespie M. N. Thannickal V. J. Langley R. J. (2020). Metabolomics to Predict Antiviral Drug Efficacy in COVID-19. Am. J. Respir. Cel. Mol. Biol. 63, 396–398. 10.1165/rcmb.2020-0206LE PMC7462337 32574504

[B163] Miller R. Wentzel A. R. Richards G. A. (2020). COVID-19: NAD+ Deficiency May Predispose the Aged, Obese and Type2 Diabetics to Mortality through its Effect on SIRT1 Activity. Med. Hypotheses 144, 110044. 10.1016/j.mehy.2020.110044 32758884 PMC7322475

[B164] Minhas P. S. Liu L. Moon P. K. Joshi A. U. Dove C. Mhatre S. (2019). Macrophage De Novo NAD+ Synthesis Specifies Immune Function in Aging and Inflammation. Nat. Immunol. 20, 50–63. 10.1038/s41590-018-0255-3 30478397 PMC6768398

[B165] Montecucco F. Bauer I. Braunersreuther V. Bruzzone S. Akhmedov A. Lüscher T. F. (2013). Inhibition of Nicotinamide Phosphoribosyltransferase Reduces Neutrophil-Mediated Injury in Myocardial Infarction. Antioxid. Redox Signaling 18, 630–641. 10.1089/ars.2011.4487 PMC3549207 22452634

[B166] Moreira D. Rodrigues V. Abengozar M. Rivas L. Rial E. Laforge M. (2015). Leishmania Infantum Modulates Host Macrophage Mitochondrial Metabolism by Hijacking the SIRT1-AMPK Axis. Plos Pathog. 11, e1004684. 10.1371/journal.ppat.1004684 25738568 PMC4349736

[B167] Morrison C. Smith G. C. M. Stingl L. Jackson S. P. Wagner E. F. Wang Z.-Q. (1997). Genetic Interaction between PARP and DNA-PK in V(D)J Recombination and Tumorigenesis. Nat. Genet. 17, 479–482. 10.1038/ng1297-479 9398855

[B168] Mukherjee P. Woods T. A. Moore R. A. Peterson K. E. (2013). Activation of the Innate Signaling Molecule Mavs by Bunyavirus Infection Upregulates the Adaptor Protein Sarm1, Leading to Neuronal Death. Immunity 38, 705–716. 10.1016/j.immuni.2013.02.013 23499490 PMC4783152

[B169] Muñoz P. Mittelbrunn M. De La Fuente H. Pérez-Martínez M. García-Pérez A. Ariza-Veguillas A. (2008). Antigen-induced Clustering of Surface CD38 and Recruitment of Intracellular CD38 to the Immunologic Synapse. Blood 111, 3653–3664. 10.1182/blood-2007-07-101600 18212246

[B170] Murray M. F. Nghiem M. Srinivasan A. (1995). HIV Infection Decreases Intracellular Nicotinamide Adenine Dinucleotide [NAD]. Biochem. Biophysical Res. Commun. 212, 126–131. 10.1006/bbrc.1995.1945 7611995

[B171] Nam T. S. Park D. R. Rah S. Y. Woo T. G. Chung H. T. Brenner C. (2020). Interleukin‐8 Drives CD38 to Form NAADP from NADP + and NAAD in the Endolysosomes to Mobilize Ca 2+ and Effect Cell Migration. FASEB j. 34, 12565–12576. 10.1096/fj.202001249R 32717131

[B172] Navarro J. Gozalbo-López B. Méndez A. C. Dantzer F. Schreiber V. Martínez C. (2017). PARP-1/PARP-2 Double Deficiency in Mouse T Cells Results in Faulty Immune Responses and T Lymphomas. Sci. Rep. 7. 10.1038/srep41962 PMC5299517 28181505

[B173] Nebenzahl-Sharon K. Sharf R. Amer J. Shalata H. Khoury-Haddad H. Sohn S.-Y. (2019). An Interaction with PARP-1 and Inhibition of Parylation Contribute to Attenuation of DNA Damage Signaling by the Adenovirus E4orf4 Protein. J. Virol. 93, 2253–2271. 10.1128/jvi.02253-18 PMC6744226 31315986

[B174] Nemoto E. Stohlman S. Dennert G. (1996). Release of a Glycosylphosphatidylinositol-Anchored ADP-Ribosyltransferase from Cytotoxic T Cells upon Activation. J. Immunol. 156, 85–92. 8598499

[B175] Nicolás L. Martínez C. Baró C. Rodríguez M. Baroja-Mazo A. Sole F. (2010). Loss of poly(ADP-Ribose) Polymerase-2 Leads to Rapid Development of Spontaneous T-Cell Lymphomas in P53-Deficient Mice. Oncogene 29, 2877–2883. 10.1038/onc.2010.11 20154718

[B176] Niere M. Mashimo M. Agledal L. Dölle C. Kasamatsu A. Kato J. (2012). ADP-ribosylhydrolase 3 (ARH3), Not poly(ADP-Ribose) Glycohydrolase (PARG) Isoforms, Is Responsible for Degradation of Mitochondrial Matrix-Associated poly(ADP-Ribose). J. Biol. Chem. 287, 16088–16102. 10.1074/jbc.M112.349183 22433848 PMC3351285

[B177] Nishizuka Y. Ueda K. Nakazawa K. Hayaishi O. (1967). Studies on the Polymer of Adenosine Diphosphate Ribose. I. Enzymic Formation from Nicotinamide Adenine Dinuclotide in Mammalian Nuclei. J. Biol. Chem. 242, 3164–3171. 10.1016/S0021-9258(18)95947-8 4291072

[B178] Nishizuka Y. Ueda K. Honjo T. Hayaishi O. (1968). Enzymic adenosine diphosphate ribosylation of histone and poly adenosine diphosphate ribose synthesis in rat liver nuclei. J. Biol. Chem. 243, 3765–3767. 10.1016/S0021-9258(19)34205-X 4298073

[B179] Nukuzuma S. Kameoka M. Sugiura S. Nakamichi K. Nukuzuma C. Takegami T. (2013). Suppressive Effect of PARP-1 Inhibitor on JC Virus Replication *In Vitro* . J. Med. Virol. 85, 132–137. 10.1002/jmv.23443 23074024

[B180] O'Connor C. M. DiMaggio P. A. Shenk T. Garcia B. A. (2014). Quantitative Proteomic Discovery of Dynamic Epigenome Changes that Control Human Cytomegalovirus (HCMV) Infection. Mol. Cell Proteomics 13, 2399–2410. 10.1074/mcp.M114.039792 24987098 PMC4159657

[B181] Oei S. L. Shi Y. (2001). Poly(adp-ribosyl)ation of Transcription Factor Yin Yang 1 under Conditions of Dna Damage. Biochem. Biophysical Res. Commun. 285, 27–31. 10.1006/bbrc.2001.5115 11437367

[B182] Okabe K. Yaku K. Tobe K. Nakagawa T. (2019). Implications of Altered NAD Metabolism in Metabolic Disorders. J. Biomed. Sci. 26. 10.1186/s12929-019-0527-8 PMC6511662 31078136

[B183] Okamoto S. Azhipa O. Yu Y. Russo E. Dennert G. (1998). Expression of ADP-Ribosyltransferase on Normal T Lymphocytes and Effects of Nicotinamide Adenine Dinucleotide on Their Function. J. Immunol. 160, 4190–4198. 9574519

[B184] Oliver F. J. Ménissier-de Murcia J. Nacci C. Decker P. Andriantsitohaina R. Muller S. (1999). Resistance to Endotoxic Shock as a Consequence of Defective NF-Kappa B Activation in Poly (ADP-Ribose) Polymerase-1 Deficient Mice. EMBO J. 18, 4446–4454. 10.1093/emboj/18.16.4446 10449410 PMC1171519

[B185] Oriol-Tordera B. Berdasco M. Llano A. Mothe B. Gálvez C. Martinez-Picado J. (2020). Methylation Regulation of Antiviral Host Factors, Interferon Stimulated Genes (ISGs) and T-Cell Responses Associated with Natural HIV Control. Plos Pathog. 16, e1008678. 10.1371/JOURNAL.PPAT.1008678 32760119 PMC7410168

[B186] Ortolan E. Augeri S. Fissolo G. Musso I. Funaro A. (2019). CD157: From Immunoregulatory Protein to Potential Therapeutic Target. Immunol. Lett. 205, 59–64. 10.1016/j.imlet.2018.06.007 29936181

[B187] Pagans S. Pedal A. North B. J. Kaehlcke K. Marshall B. L. Dorr A. (2005). SIRT1 Regulates HIV Transcription via Tat Deacetylation. Plos Biol. 3, e41. 10.1371/journal.pbio.0030041 15719057 PMC546329

[B188] Pajuelo D. Gonzalez-Juarbe N. Tak U. Sun J. Orihuela C. J. Niederweis M. (2018). NAD+ Depletion Triggers Macrophage Necroptosis, a Cell Death Pathway Exploited by *Mycobacterium tuberculosis* . Cel Rep. 24, 429–440. 10.1016/j.celrep.2018.06.042 PMC6136256 29996103

[B189] Panneerselvam P. Singh L. P. Selvarajan V. Chng W. J. Ng S. B. Tan N. S. (2013). T-cell Death Following Immune Activation Is Mediated by Mitochondria-Localized SARM. Cel. Death Differ 20, 478–489. 10.1038/cdd.2012.144 PMC3569988 23175186

[B190] Paone G. Stevens L. A. Levine R. L. Bourgeois C. Steagall W. K. Gochuico B. R. (2006). ADP-ribosyltransferase-specific Modification of Human Neutrophil Peptide-1. J. Biol. Chem. 281, 17054–17060. 10.1074/jbc.M603042200 16627471

[B191] Paone G. Wada A. Stevens L. A. Matin A. Hirayama T. Levine R. L. (2002). ADP Ribosylation of Human Neutrophil Peptide-1 Regulates its Biological Properties. Proc. Natl. Acad. Sci. 99, 8231–8235. 10.1073/PNAS.122238899 12060767 PMC123050

[B192] Pellat-Deceunynck C. Wietzerbin J. Drapier J. C. (1994). Nicotinamide Inhibits Nitric Oxide Synthase mRNA Induction in Activated Macrophages. Biochem. J. 297, 53–58. 10.1042/bj2970053 7506533 PMC1137789

[B193] Peng J. Yuan Q. Lin B. Panneerselvam P. Wang X. Luan X. L. (2010). SARM Inhibits Both TRIF- and MyD88-Mediated AP-1 Activation. Eur. J. Immunol. 40, 1738–1747. 10.1002/eji.200940034 20306472

[B194] Pfluger P. T. Herranz D. Velasco-Miguel S. Serrano M. Tschöp M. H. (2008). Sirt1 Protects against High-Fat Diet-Induced Metabolic Damage. Proc. Natl. Acad. Sci. 105, 9793–9798. 10.1073/pnas.0802917105 18599449 PMC2474520

[B195] Piekna-Przybylska D. Nagumotu K. Reid D. M. Maggirwar S. B. (2019). HIV-1 Infection Renders Brain Vascular Pericytes Susceptible to the Extracellular Glutamate. J. Neurovirol. 25, 114–126. 10.1007/s13365-018-0693-6 30402824 PMC6417930

[B196] Planavila A. Iglesias R. Giralt M. Villarroya F. (2011). Sirt1 Acts in Association with PPAR to Protect the Heart from Hypertrophy, Metabolic Dysregulation, and Inflammation. Cardiovasc. Res. 90, 276–284. 10.1093/cvr/cvq376 21115502

[B197] Preyat N. Leo O. (2013). Sirtuin Deacylases: a Molecular Link between Metabolism and Immunity. J. Leukoc. Biol. 93, 669–680. 10.1189/jlb.1112557 23325925

[B198] Pudla M. Limposuwan K. Utaisincharoen P. (2011). Burkholderia Pseudomallei-Induced Expression of a Negative Regulator, Sterile-α and Armadillo Motif-Containing Protein, in Mouse Macrophages: a Possible Mechanism for Suppression of the MyD88-independent Pathway. Infect. Immun. 79, 2921–2927. 10.1128/IAI.01254-10 21555400 PMC3191974

[B199] Qi L. Hu M. Fu J. Liu Y. Wu M. Yu K. (2017). Quantitative Proteomic Analysis of Host Epithelial Cells Infected bySalmonella Entericaserovar Typhimurium. Proteomics 17, 1700092. 10.1002/pmic.201700092 28544771

[B200] Rack J. G. M. Zorzini V. Zhu Z. Schuller M. Ahel D. Ahel I. (2020). Viral Macrodomains: A Structural and Evolutionary Assessment of the Pharmacological Potential. Open Biol. 10, 200237. 10.1098/rsob.200237 33202171 PMC7729036

[B201] Raffaelli N. Sorci L. Amici A. Emanuelli M. Mazzola F. Magni G. (2002). Identification of a Novel Human Nicotinamide Mononucleotide Adenylyltransferase. Biochem. Biophys. Res. Commun. 297, 835–840. 10.1016/S0006-291X(02)02285-4 12359228

[B202] Ren J.-H. Tao Y. Zhang Z.-Z. Chen W.-X. Cai X.-F. Chen K. (2014). Sirtuin 1 Regulates Hepatitis B Virus Transcription and Replication by Targeting Transcription Factor AP-1. J. Virol. 88, 2442–2451. 10.1128/jvi.02861-13 24335313 PMC3958108

[B203] Ren J. H. Hu J. L. Cheng S. T. Yu H. B. Wong V. K. W. Law B. Y. K. (2018). SIRT3 Restricts Hepatitis B Virus Transcription and Replication through Epigenetic Regulation of Covalently Closed Circular DNA Involving Suppressor of Variegation 3‐9 Homolog 1 and SET Domain Containing 1A Histone Methyltransferases. Hepatology 68, 1260–1276. 10.1002/hep.29912 29624717

[B204] Revollo J. R. Grimm A. A. Imai S.-i. (2004). The NAD Biosynthesis Pathway Mediated by Nicotinamide Phosphoribosyltransferase Regulates Sir2 Activity in Mammalian Cells. J. Biol. Chem. 279, 50754–50763. 10.1074/jbc.M408388200 15381699

[B205] Rissiek B. Menzel S. Leutert M. Cordes M. Behr S. Jank L. (2017). Ecto-ADP-ribosyltransferase ARTC2.1 Functionally Modulates FcγR1 and FcγR2B on Murine Microglia. Sci. Rep. 7. 10.1038/S41598-017-16613-W PMC5705771 29184112

[B206] Robinson N. Ganesan R. Hegedűs C. Kovács K. Kufer T. A. Virág L. (2019). Programmed Necrotic Cell Death of Macrophages: Focus on Pyroptosis, Necroptosis, and Parthanatos. Redox Biol. 26, 101239. 10.1016/j.redox.2019.101239 31212216 PMC6582207

[B207] Rodríguez‐Alba J. C. Abrego‐Peredo A. Gallardo‐Hernández C. Pérez‐Lara J. Santiago‐Cruz W. Jiang W. (2019). HIV Disease Progression: Overexpression of the Ectoenzyme CD38 as a Contributory Factor? BioEssays 41, e1800128. 10.1002/bies.201800128 30537007 PMC6545924

[B208] Rom S. Reichenbach N. L. Dykstra H. Persidsky Y. (2015). The Dual Action of poly(ADP-Ribose) Polymerase -1 (PARP-1) Inhibition in HIV-1 Infection: HIV-1 Ltr Inhibition and Diminution in Rho GTPase Activity. Front. Microbiol. 6. 10.3389/fmicb.2015.00878 PMC4548080 26379653

[B209] Roy S. Liu F. Arav-Boger R. (2015). Human Cytomegalovirus Inhibits the PARsylation Activity of Tankyrase-A Potential Strategy for Suppression of the Wnt Pathway. Viruses 8, 8. 10.3390/v8010008 PMC4728568 26729153

[B210] Rudolph J. Roberts G. Muthurajan U. M. Luger K. (2021). HPF1 and Nucleosomes Mediate a Dramatic Switch in Activity of PARP1 from Polymerase to Hydrolase. Elife 10. 10.7554/elife.65773 PMC8012059 33683197

[B211] Ruiz P. D. Hamilton G. A. Park J. W. Gamble M. J. (2019). MacroH2A1 Regulation of Poly(ADP-Ribose) Synthesis and Stability Prevents Necrosis and Promotes DNA Repair. Mol. Cell. Biol. 40. 10.1128/mcb.00230-19 PMC6908255 31636161

[B212] Ryu K. W. Nandu T. Kim J. Challa S. DeBerardinis R. J. Kraus W. L. (2018). Metabolic Regulation of Transcription through Compartmentalized NAD+biosynthesis. Science 360, eaan5780. 10.1126/science.aan5780 29748257 PMC6465534

[B213] Sallard E. Lescure F.-X. Yazdanpanah Y. Mentre F. Peiffer-Smadja N. (2020). Type 1 Interferons as a Potential Treatment against COVID-19. Antiviral Res. 178, 104791. 10.1016/j.antiviral.2020.104791 32275914 PMC7138382

[B214] Savarino A. Bottarel F. Malavasi F. Dianzani U. (2000). Role of CD38 in HIV-1 Infection: An Epiphenomenon of T-Cell Activation or an Active Player in Virus/host Interactions? AIDS 14, 1079–1089. 10.1097/00002030-200006160-00004 10894271

[B215] Saxty B. A. Yadollahi-Farsani M. Upton P. D. Johnstone S. R. MacDermot J. (2001). Inactivation of Platelet-Derived Growth Factor-BB Following Modification by ADP-Ribosyltransferase. Br. J. Pharmacol. 133, 1219–1226. 10.1038/sj.bjp.0704187 11498506 PMC1621139

[B216] Schiavoni I. Scagnolari C. Horenstein A. L. Leone P. Pierangeli A. Malavasi F. (2018). CD38 Modulates Respiratory Syncytial Virus-Driven Proinflammatory Processes in Human Monocyte-Derived Dendritic Cells. Immunology 154, 122–131. 10.1111/imm.12873 29178427 PMC5904717

[B217] Schilling E. Wehrhahn J. Klein C. Raulien N. Ceglarek U. Hauschildt S. (2012). Inhibition of Nicotinamide Phosphoribosyltransferase Modifies LPS-Induced Inflammatory Responses of Human Monocytes. Innate Immun. 18, 518–530. 10.1177/1753425911423853 21975728

[B218] Schuller M. Correy G. J. Gahbauer S. Fearon D. Wu T. Díaz R. E. (2021). SARS-CoV-2 Identified through Crystallographic Screening and Computational Docking. Sci. Adv. 7, 8711–8725. 10.1126/sciadv.abf8711 PMC8046379 33853786

[B219] Schweiker S. S. Tauber A. L. Sherry M. E. Levonis S. M. (2018). Structure, Function and Inhibition of Poly(ADP-Ribose)polymerase, Member 14 (PARP14). Mrmc 18, 1659–1669. 10.2174/1389557518666180816111749 30112992

[B220] Schwerk J. Soveg F. W. Ryan A. P. Thomas K. R. Hatfield L. D. Ozarkar S. (2019). RNA-binding Protein Isoforms ZAP-S and ZAP-L Have Distinct Antiviral and Immune Resolution Functions. Nat. Immunol. 20, 1610–1620. 10.1038/s41590-019-0527-6 31740798 PMC7240801

[B221] Seki M. Fairchild S. Rosenwasser O. A. Tada N. Tomonari K. (2001). An Immature Rat Lymphocyte Marker CD157: Striking Differences in the Expression between Mice and Rats. Immunobiology 203, 725–742. 10.1016/S0171-2985(01)80002-4 11563673

[B222] Seman M. Adriouch S. Scheuplein F. Krebs C. Freese D. Glowacki G. (2003). NAD-induced T Cell Death. Immunity 19, 571–582. 10.1016/S1074-7613(03)00266-8 14563321

[B223] Shockett P. Stavnezer J. (1993). Inhibitors of poly(ADP-Ribose) Polymerase Increase Antibody Class Switching. J. Immunol. 151, 6962–6976. 8258703

[B224] Shou Q. Fu H. Huang X. Yang Y. (2019). PARP-1 Controls NK Cell Recruitment to the Site of Viral Infection. JCI Insight 4. 10.1172/jci.insight.121291 PMC6629106 31217354

[B225] Siva A. C. Bushman F. (2002). Poly(ADP-Ribose) Polymerase 1 Is Not Strictly Required for Infection of Murine Cells by Retroviruses. J. Virol. 76, 11904–11910. 10.1128/jvi.76.23.11904-11910.2002 12414932 PMC136881

[B226] Sun L. J. Yu J. W. Shi Y. G. Zhang X. Y. Shu M. N. Chen M. Y. (2018). Hepatitis C Virus Core Protein Induces Dysfunction of Liver Sinusoidal Endothelial Cell by Down‐regulation of Silent Information Regulator 1. J. Med. Virol. 90, 926–935. 10.1002/jmv.25034 29350417

[B227] Suskiewicz M. J. Zobel F. Ogden T. E. H. Fontana P. Ariza A. Yang J.-C. (2020). HPF1 Completes the PARP Active Site for DNA Damage-Induced ADP-Ribosylation. Nature 579, 598–602. 10.1038/s41586-020-2013-6 32028527 PMC7104379

[B228] Szabó C. Lim L. H. K. Cuzzocrea S. Getting S. J. Zingarelli B. Flower R. J. (1997). Inhibition of Poly (ADP-Ribose) Synthetase Attenuates Neutrophil Recruitment and Exerts Antiinflammatory Effects. J. Exp. Med. 186, 1041–1049. 10.1084/jem.186.7.1041 9314553 PMC2199068

[B229] Szretter K. J. Samuel M. A. Gilfillan S. Fuchs A. Colonna M. Diamond M. S. (2009). The Immune Adaptor Molecule SARM Modulates Tumor Necrosis Factor Alpha Production and Microglia Activation in the Brainstem and Restricts West Nile Virus Pathogenesis. J. Virol. 83, 9329–9338. 10.1128/jvi.00836-09 19587044 PMC2738257

[B230] Tang Q. Wang X. Gao G. (2017). The Short Form of the Zinc Finger Antiviral Protein Inhibits Influenza A Virus Protein Expression and Is Antagonized by the Virus-Encoded NS1. J. Virol. 91. 10.1128/jvi.01909-16 PMC5215320 27807230

[B231] Tanny J. C. Dowd G. J. Huang J. Hilz H. Moazed D. (1999). An Enzymatic Activity in the Yeast Sir2 Protein that Is Essential for Gene Silencing. Cell 99, 735–745. 10.1016/S0092-8674(00)81671-2 10619427

[B232] Tarragó M. G. Chini C. C. S. Kanamori K. S. Warner G. M. Caride A. de Oliveira G. C. (2018). A Potent and Specific CD38 Inhibitor Ameliorates Age-Related Metabolic Dysfunction by Reversing Tissue NAD+ Decline. Cel Metab. 27, 1081–1095. 10.1016/j.cmet.2018.03.016 PMC5935140 29719225

[B233] Teixeira C. S. S. Cerqueira N. M. F. S. A. Gomes P. Sousa S. F. (2020). A Molecular Perspective on Sirtuin Activity. Ijms 21, 8609–8620. 10.3390/ijms21228609 PMC7696986 33203121

[B234] Tempera I. Deng Z. Atanasiu C. Chen C.-J. D'Erme M. Lieberman P. M. (2010). Regulation of Epstein-Barr Virus OriP Replication by Poly(ADP-Ribose) Polymerase 1. J. Virol. 84, 4988–4997. 10.1128/jvi.02333-09 20219917 PMC2863838

[B235] Thormar H. Isaacs C. E. Brown H. R. Barshatzky M. R. Pessolano T. (1987). Inactivation of Enveloped Viruses and Killing of Cells by Fatty Acids and Monoglycerides. Antimicrob. Agents Chemother. 31, 27–31. 10.1128/AAC.31.1.27 3032090 PMC174645

[B236] Todd R. F. Roach J. A. Arnaout M. A. (1985). The Modulated Expression of Mo5, a Human Myelomonocytic Plasma Membrane Antigen. Blood 65, 964–973. 10.1182/blood.V65.4.964.964 2579692

[B237] Tong L. Denu J. M. (2010). Function and Metabolism of Sirtuin Metabolite O-Acetyl-ADP-Ribose. Biochim. Biophys. Acta (Bba) - Proteins Proteomics 1804, 1617–1625. 10.1016/j.bbapap.2010.02.007 20176146 PMC3310390

[B238] Ueda K. Reeder R. H. Honjo T. Nishizuka Y. Hayaishi O. (1968). Poly Adenosine Diphosphate Ribose Synthesis Associated with Chromatin. Biochem. Biophysical Res. Commun. 31, 379–385. 10.1016/0006-291X(68)90486-5 5653649

[B239] Valdor R. Schreiber V. Saenz L. Martínez T. Muñoz-Suano A. Dominguez-Villar M. (2008). Regulation of NFAT by poly(ADP-Ribose) Polymerase Activity in T Cells. Mol. Immunol. 45, 1863–1871. 10.1016/j.molimm.2007.10.044 18078995

[B240] Van Gool F. Gallí M. Gueydan C. Kruys V. Prevot P.-P. Bedalov A. (2009). Intracellular NAD Levels Regulate Tumor Necrosis Factor Protein Synthesis in a Sirtuin-dependent Manner. Nat. Med. 15, 206–210. 10.1038/nm.1906 19151729 PMC2845476

[B241] VanLinden M. R. Dölle C. Pettersen I. K. N. Kulikova V. A. Niere M. Agrimi G. (2015). Subcellular Distribution of NAD+ between Cytosol and Mitochondria Determines the Metabolic Profile of Human Cells. J. Biol. Chem. 290, 27644–27659. 10.1074/jbc.M115.654129 26432643 PMC4646015

[B242] Vastag L. Koyuncu E. Grady S. L. Shenk T. E. Rabinowitz J. D. (2011). Divergent Effects of Human Cytomegalovirus and Herpes Simplex Virus-1 on Cellular Metabolism. Plos Pathog. 7, e1002124. 10.1371/journal.ppat.1002124 21779165 PMC3136460

[B243] Viar K. Njoku D. Secor McVoy J. Oh U. (2020). Sarm1 Knockout Protects against Early but Not Late Axonal Degeneration in Experimental Allergic Encephalomyelitis. PLoS One 15, e0235110. 10.1371/journal.pone.0235110 32584865 PMC7316289

[B244] Virág L. Salzman A. L. Szabó C. (1998). Poly(ADP-ribose) Synthetase Activation Mediates Mitochondrial Injury during Oxidant-Induced Cell Death. J. Immunol. 161, 3753–3759. 9759901

[B245] Vyas S. Matic I. Uchima L. Rood J. Zaja R. Hay R. T. (2014). Family-wide Analysis of poly(ADP-Ribose) Polymerase Activity. Nat. Commun. 5. 10.1038/ncomms5426 PMC4123609 25043379

[B246] Wang X. Sun B. Mbondji C. Biswas S. Zhao J. Hewlett I. (2017). Differences in Activation of HIV-1 Replication by Superinfection with HIV-1 and HIV-2 in U1 Cells. J. Cell. Physiol. 232, 1746–1753. 10.1002/jcp.25614 27662631

[B247] Wei W. Graeff R. Yue J. (2014). Roles and Mechanisms of the CD38/cyclic Adenosine Diphosphate ribose/Ca2+signaling Pathway. Wjbc 5, 58. 10.4331/wjbc.v5.i1.58 24600514 PMC3942542

[B248] Weixler L. Schäringer K. Momoh J. Lüscher B. Feijs K. L. H. Žaja R. (2021). ADP-ribosylation of RNA and DNA: from *In Vitro* Characterization to *In Vivo* Function. Nucleic Acids Res. 49, 3634–3650. 10.1093/nar/gkab136 33693930 PMC8053099

[B249] Westera L. Jennings A. M. Maamary J. Schwemmle M. García-Sastre A. Bortz E. (2019). Poly-ADP Ribosyl Polymerase 1 (PARP1) Regulates Influenza A Virus Polymerase. Adv. Virol. 2019, 1–11. 10.1155/2019/8512363 PMC6444269 31015836

[B250] Wonigeit K. Dinkel A. Fangmann J. Thude H. (1997). Expression of the Ectoenzyme RT6 Is Not Restricted to Resting Peripheral T Cells and Is Differently Regulated in normal Peripheral T Cells, Intestinal IEL, and NK Cells. Adv. Exp. Med. Biol. 419, 229–240. 10.1007/978-1-4419-8632-0_28 9193658

[B251] Xander N. Reddy Vari H. Eskandar R. Li W. Bolla S. Marchetti N. (2019). Rhinovirus-Induced SIRT-1 via TLR2 Regulates Subsequent Type I and Type III IFN Responses in Airway Epithelial Cells. J.I. 203, 2508–2519. 10.4049/jimmunol.1900165 PMC6810856 31548332

[B252] Xia C. Wolf J. J. Sun C. Xu M. Studstill C. J. Chen J. (2020). PARP1 Enhances Influenza A Virus Propagation by Facilitating Degradation of Host Type I Interferon Receptor. J. Virol. 94. 10.1128/jvi.01572-19 PMC7081902 31915279

[B253] Xiao W. Wang R.-S. Handy D. E. Loscalzo J. (2018). NAD(H) and NADP(H) Redox Couples and Cellular Energy Metabolism. Antioxid. Redox Signaling 28, 251–272. 10.1089/ars.2017.7216 PMC5737637 28648096

[B254] Xie H. Lei N. Gong A.-Y. Chen X.-M. Hu G. (2014). Cryptosporidium Parvum Induces SIRT1 Expression in Host Epithelial Cells through Downregulating Let-7i. Hum. Immunol. 75, 760–765. 10.1016/j.humimm.2014.05.007 24862934 PMC4327856

[B255] Xie L. Lu B. Zheng Z. Miao Y. Liu Y. Zhang Y. (2018). The 3C Protease of Enterovirus A71 Counteracts the Activity of Host Zinc-finger Antiviral Protein (ZAP). J. Gen. Virol. 99, 73–85. 10.1099/jgv.0.000982 29182509

[B256] Xu G. Li S. Liu X. Gao P. Chen X. Wang H. (2019). PARP-1 Mediated Cell Death Is Directly Activated by ZIKV Infection. Virology 537, 254–262. 10.1016/j.virol.2019.08.024 31539774

[B257] Yamada T. Horimoto H. Kameyama T. Hayakawa S. Yamato H. Dazai M. (2016). Constitutive Aryl Hydrocarbon Receptor Signaling Constrains Type I Interferon-Mediated Antiviral Innate Defense. Nat. Immunol. 17, 687–694. 10.1038/ni.3422 27089381

[B258] Yamai T. Hikita H. Fukuoka M. Fukutomi K. Murai K. Nakabori T. (2020). SIRT1 Enhances Hepatitis Virus B Transcription Independent of Hepatic Autophagy. Biochem. Biophysical Res. Commun. 527, 64–70. 10.1016/j.bbrc.2020.04.031 32446392

[B259] Yang C.-S. Jividen K. Spencer A. Dworak N. Ni L. Oostdyk L. T. (2017). Ubiquitin Modification by the E3 Ligase/ADP-Ribosyltransferase Dtx3L/Parp9. Mol. Cel. 66, 503–516. 10.1016/j.molcel.2017.04.028 PMC5556935 28525742

[B260] Yang H. Zhang W. Pan H. Feldser H. G. Lainez E. Miller C. (2012). SIRT1 Activators Suppress Inflammatory Responses through Promotion of P65 Deacetylation and Inhibition of NF-Κb Activity. PLoS One 7, e46364. 10.1371/journal.pone.0046364 23029496 PMC3460821

[B261] Yang Q. Liao M. Wang W. Zhang M. Chen Q. Guo J. (2019). CD157 Confers Host Resistance to *Mycobacterium tuberculosis* via TLR2-Cd157-PKCzeta-Induced Reactive Oxygen Species Production. MBio 10. 10.1128/mBio.01949-19 PMC6712401 31455656

[B262] Yang Z. Kahn B. B. Shi H. Xue B.-z. (2010). Macrophage α1 AMP-Activated Protein Kinase (α1AMPK) Antagonizes Fatty Acid-Induced Inflammation through SIRT1. J. Biol. Chem. 285, 19051–19059. 10.1074/jbc.M110.123620 20421294 PMC2885183

[B263] Yélamos J. Monreal Y. Saenz L. Aguado E. Schreiber V. Mota R. (2006). PARP-2 Deficiency Affects the Survival of CD4+CD8+ Double-Positive Thymocytes. EMBO J. 25, 4350–4360. 10.1038/sj.emboj.7601301 16946705 PMC1570435

[B264] Yeung F. Hoberg J. E. Ramsey C. S. Keller M. D. Jones D. R. Frye R. A. (2004). Modulation of NF-κb-dependent Transcription and Cell Survival by the SIRT1 Deacetylase. EMBO J. 23, 2369–2380. 10.1038/sj.emboj.7600244 15152190 PMC423286

[B265] Ying W. (2008). NAD+/NADH and NADP+/NADPH in Cellular Functions and Cell Death: Regulation and Biological Consequences. Antioxid. Redox Signaling 10, 179–206. 10.1089/ars.2007.1672 18020963

[B266] Yu D. Liu R. Yang G. Zhou Q. (2018a). The PARP1-Siah1 Axis Controls HIV-1 Transcription and Expression of Siah1 Substrates. Cel. Rep. 23, 3741–3749. 10.1016/j.celrep.2018.05.084 PMC6223328 29949759

[B267] Yu H.-B. Jiang H. Cheng S.-T. Hu Z.-W. Ren J.-H. Chen J. (2018b). AGK2, A SIRT2 Inhibitor, Inhibits Hepatitis B Virus Replication *In Vitro* and *In Vivo* . Int. J. Med. Sci. 15, 1356–1364. 10.7150/IJMS.26125 30275764 PMC6158674

[B268] Yu J.-W. Sun L.-J. Liu W. Zhao Y.-H. Kang P. Yan B.-Z. (2013). Hepatitis C Virus Core Protein Induces Hepatic Metabolism Disorders through Down-Regulation of the SIRT1-AMPK Signaling Pathway. Int. J. Infect. Dis. 17, e539–e545. 10.1016/j.ijid.2013.01.027 23510538

[B269] Yu M. Zhang C. Yang Y. Yang Z. Zhao L. Xu L. (2011). The Interaction between the PARP10 Protein and the NS1 Protein of H5N1 AIV and its Effect on Virus Replication. Virol. J. 8, 546. 10.1186/1743-422X-8-546 22176891 PMC3287249

[B270] Yu Q. Dong L. Li Y. Liu G. (2018c). SIRT1 and HIF1α Signaling in Metabolism and Immune Responses. Cancer Lett. 418, 20–26. 10.1016/j.canlet.2017.12.035 29306019

[B271] Yuan H. Marmorstein R. (2012). Structural Basis for Sirtuin Activity and Inhibition. J. Biol. Chem. 287, 42428–42435. 10.1074/jbc.R112.372300 23086949 PMC3522243

[B272] Zapata‐Pérez R. Tammaro A. Schomakers B. V. Scantlebery A. M. L. Denis S. Elfrink H. L. (2021). Reduced Nicotinamide Mononucleotide Is a New and Potent NAD + Precursor in Mammalian Cells and Mice. FASEB j. 35. 10.1096/fj.202001826R 33724555

[B273] Zerez C. Roth E. J. Schulman S. Tanaka K. (1990). Increased Nicotinamide Adenine Dinucleotide Content and Synthesis in Plasmodium Falciparum-Infected Human Erythrocytes. Blood 75, 1705–1710. 10.1182/blood.v75.8.1705.170510.1182/blood.v75.8.1705.bloodjournal7581705 2183889

[B274] Zhang C. Wang J. Zhang H. Liu S. Lee H. J. Jin W. (2018). Hepatitis C Virus Core Protein Induces Hepatic Steatosis via Sirt1-dependent Pathway. Liver Int. 38, 803–812. 10.1111/liv.13581 28898508

[B275] Zhang C. Wang K. Hu Z. Yang L. Wei B. Li S. (2020). SIRT5 Is Important for Bacterial Infection by Regulating Insulin Secretion and Glucose Homeostasis. Protein Cell 11, 846–851. 10.1007/s13238-020-00709-7 32415480 PMC7647967

[B276] Zhang J. Tao J. Ling Y. Li F. Zhu X. Xu L. (2019). Switch of NAD Salvage to De Novo Biosynthesis Sustains SIRT1-RelB-dependent Inflammatory Tolerance. Front. Immunol. 10. 10.3389/fimmu.2019.02358 PMC6797595 31681271

[B277] Zhang Y. Mao D. Roswit W. T. Jin X. Patel A. C. Patel D. A. (2015). PARP9-DTX3L Ubiquitin Ligase Targets Host Histone H2BJ and Viral 3C Protease to Enhance Interferon Signaling and Control Viral Infection. Nat. Immunol. 16, 1215–1227. 10.1038/ni.3279 26479788 PMC4653074

[B278] Zhao Y. Song Z. Bai J. Liu X. Nauwynck H. Jiang P. (2019). ZAP, a CCCH-type Zinc Finger Protein, Inhibits Porcine Reproductive and Respiratory Syndrome Virus Replication and Interacts with Viral Nsp9. J. Virol. 93. 10.1128/jvi.00001-19 PMC6498049 30867303

[B279] Zhou T. Kurnasov O. Tomchick D. R. Binns D. D. Grishin N. V. Marquez V. E. (2002). Structure of Human Nicotinamide/Nicotinic Acid Mononucleotide Adenylyltransferase. J. Biol. Chem. 277, 13148–13154. 10.1074/jbc.M111469200 11788603

[B280] Zielinska W. Barata H. Chini E. N. (2004). Metabolism of Cyclic ADP-Ribose: Zinc Is an Endogenous Modulator of the Cyclase/NAD Glycohydrolase Ratio of a CD38-like Enzyme from Human Seminal Fluid. Life Sci. 74, 1781–1790. 10.1016/j.lfs.2003.08.033 14741735

[B281] Zolkiewska A. Moss J. (1993). Integrin Alpha 7 as Substrate for a Glycosylphosphatidylinositol-Anchored ADP-Ribosyltransferase on the Surface of Skeletal Muscle Cells. J. Biol. Chem. 268, 25273–25276. 10.1016/S0021-9258(19)74388-9 8244957

[B282] Zolkiewska A. Nightingale M. S. Moss J. (1992). Molecular Characterization of NAD: arginine ADP-Ribosyltransferase from Rabbit Skeletal Muscle. Proc. Natl. Acad. Sci. 89, 11352–11356. 10.1073/pnas.89.23.11352 1454819 PMC50548

